# Assessing the Impact of Nanoplastics in Biological Systems: Systematic Review of In Vitro Animal Studies

**DOI:** 10.3390/jox15030075

**Published:** 2025-05-17

**Authors:** Maria Viana, Fernanda S. Tonin, Carina Ladeira

**Affiliations:** 1 ESTeSL-Escola Superior de Tecnologia da Saúde, Instituto Politécnico de Lisboa, 1990-096 Lisbon, Portugal; marsea.rlv@gmail.com; 2 H&TRC-Health & Technology Research Center, ESTeSL-Escola Superior de Tecnologia da Saúde, Instituto Politécnico de Lisboa, 1990-096 Lisbon, Portugal; ffstonin@gmail.com; 3 Pharmacy and Pharmaceutical Technology Department, Social and Legal Pharmacy Section, University of Granada, 18071 Granada, Spain; 4 NOVA National School of Public Health, Public Health Research Centre, Universidade NOVA de Lisboa, 1600-560 Lisbon, Portugal

**Keywords:** nanoplastics, in vitro, cellular lines, cytotoxicity, organ system, systematic review

## Abstract

Nanoplastic (NP) pollution has emerged as a growing concern due to its potential impact on human health, although its adverse effects on different organ systems are not yet fully understood. This systematic scoping review, conducted in accordance with international guidelines, aimed to map the current evidence on the biological effects of NPs. In vitro animal studies assessing cellular damage caused by exposure to any type of NP were searched on PubMed, Web of Science, and Scopus. Data on primary outcomes related to genotoxicity and cytotoxicity (cell viability, oxidative stress, inflammation, DNA and cytoplasmic damage, apoptosis) were extracted from the included studies, and overall reporting quality was assessed. A total of 108 articles published between 2018 and 2024, mostly by China (54%), Spain (14%), and Italy (9%), were included. Polystyrene (PS) was the most frequently studied polymer (85%). NP sizes in solution ranged from 15 to 531 nm, with a higher prevalence in the 40–100 nm range (38%). The overall quality of studies was rated as moderate (60%), with many lacking essential details about cell culture conditions (e.g., pH of the medium, passage number, substances used). A higher frequency of negative effects from NP exposure was observed in respiratory cell lines, while immune, digestive, and hepatic cell lines showed greater resistance. Nervous, urinary, and connective tissue systems were impacted by NPs. Positively charged and smaller PS particles were consistently associated with higher toxicity across all systems. In summary, this review highlights the multifactorial nature of NP toxicity, influenced by size, surface charge, and polymer type. It also reveals a significant knowledge gap, stemming from the predominant use of immortalized monocultures exposed to commercially available PS NPs, the limited use of environmentally relevant particles, and the underutilization of advanced experimental models (e.g., organ-on-chip systems) that better mimic physiological conditions.

## 1. Introduction

Plastics play a crucial role in various industries, including transportation, food, healthcare, and energy, contributing significantly to modern life. Synthetic items, such as micro- and nanoplastics (MNPs), exhibit resilience, low weight, resistance to deterioration, robustness, affordability, and adeptness in insulating against heat and electricity, thus having widespread utilization worldwide [1,2,3].

The European Chemicals Agency (ECHA) estimates that each year, around 42,000 tons of intentionally added microplastics end up in the environment [4]. MNPs are small polymeric particles primarily distinguished by their scale [5]. According to some authors, microplastics (MPs) have dimensions between 1 µm and 5 mm [6], while others classify them as being under 5 mm [7,8]. As for nanoplastics (NPs), they are mostly either considered to range from 1 to 100 nm [9,10] or from 1 to 1000 nm [6,7], depending on the author or guidelines. However other definitions for plastics according to different size distributions continue to exist in the current literature [11,12,13]. The definition and classification criteria for NPs have been reviewed, discussed, and proposed, remaining subject to refinement as the field progresses [14,15]. Nonetheless, the most used ranges are 1–5 µm for MPs and 1–1000 nm for NPs. In this study, NPs are considered based on the metric scale and defined as particles below 1000 nm.

Plastic’s stability and durability prevents it from degrading or breaking down over time, and when coupled with rising manufacturing numbers leads to the projection that an additional 33 billion tons of plastic will have accumulated on the planet by 2050 [16]. Results from 2015, nearly 80% of the plastic waste generated ended up in either landfill or the environment, while only 9% was recycled [17], and it is suggested that by 2050, another 33 billion tons of plastic will have accumulated on the planet. When combined with the awareness of plastic’s established and potential toxicity, these statistics sound like a compelling alarm, urging immediate action to mitigate pollution across nations [16].

**Figure 1:**
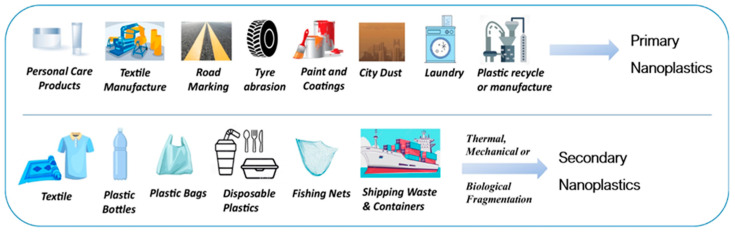
Common sources of primary and secondary NPs. Adapted from [20].

Over 80% of plastic debris originates from land-based sources, with less than 20% originating from sea-based activities [18,19]. Broadly, sources of NPs can be categorized into primary and secondary (Figure 1) [20], contingent on their formation mechanisms [21,22].

Primary NPs are intentionally manufactured as raw materials [9] and are widely used in cosmetics formulations, personal care products containing scrubs and abrasives, paints, industrial abrasives, industrial air-blasting, filaments for 3D printing, and drug vectors. NPs originating during manufacturing or utilization of plastic products, such as tire abrasion, road markings, marine coatings, textiles, and laundry, are also included in this category [23,24].

Secondary NPs can result from the weathering and fragmentation of larger plastic items [19,25,26]. This breakdown may occur through several processes, namely chemical (photodegradation), physical (mechanical abrasion), and biological (biodegradation by microbial species) [27]. The presence of secondary NPs in the environment is proportional to the population, number of vehicles, and laundering operations [28].

NPs exhibit various behaviors in different environmental media, such as air, water, soil, and biota. NPs can be transported through the air from sources like industrial activities [29,30], traffic [31], or plastic particles from textiles [32]. Once in the air, they can travel over long distances and potentially settle on surfaces or be deposited into bodies of water, soil, or ecosystems through atmospheric deposition [33]. NPs can enter water bodies through various pathways, such as runoff from land, wastewater discharge, or direct release [34,35]. NPs can also interact with organisms in water, potentially leading to ingestion by aquatic species [36]. NPs can reach soil through various routes, including runoff from land, application of plastic-containing products like mulches or fertilizers, or direct deposition [37]. Once in the soil, they can undergo processes like adsorption to soil particles, transportation through soil pores, or interaction with soil microorganisms, being able to affect plant and soil health [38]. Finally, NPs can be ingested by different organisms and affect species at a higher trophic level due to being retained and trophically transferred across the food chain. Once ingested, NPs can potentially accumulate in tissues and organs, leading to adverse effects such as inflammation, oxidative stress, or disruption of physiological processes [39]. NPs can also transfer through food webs, potentially impacting entire ecosystems [40].

NPs are particularly problematic due to their small dimensions, which enable them to travel further and more widely in the environment, raising concerns about their pervasiveness. Furthermore, their diminutive dimensions allow them to interact with biological systems at organic and cellular levels. These particles can cross the cytoplasmic membrane of cells and interact with cellular organelles, leading to genotoxicity, cytotoxicity, or other forms of cellular damage [41]. Modifications to their chemical surface, created by weathering processes or present since manufacturing, can enhance their potential for cellular damage, with certain surface chemistries being more harmful than others [42]. In particular, the capability to disrupt metabolic processes raises significant concerns about their impact on human health [43]. Understanding the effects of NPs on biological systems is therefore essential for assessing their potential hazards and informing regulatory decisions.

However, despite growing concern, knowledge about the real impact of NPs on human health remains limited. Most studies rely on a narrow set of model polymers [5,44,45,46], and often use high concentrations that do not reflect realistic exposure scenarios [46,47,48,49]. Furthermore, some research tends to use simple monocultures that lack the complexity of human tissues, while in vivo studies are still scarce and often do not simulate chronic, low-dose exposures [10,11,50,51,52,53,54]. The lack of standardized methodologies for detection, quantification, and characterization of NPs further complicates efforts to assess their biological effects and environmental behavior [53,54].

In this context, we aimed to synthesize the available evidence on the effects of NPs in biological systems, assessed through in vitro animal studies, and describe whenever possible the different impacts of these substances according to their size, polymer type, and cell type.

## 2. Materials and Methods

### 2.1. Searches and Eligibility Criteria

A broad systematic scoping review following *JBI Manual for Evidence Synthesis* [55] and reported according to the PRISMA ScR extension for scoping reviews [56] was performed to map the evidence on the effects of NPs. Two independent reviewers conducted all the main steps of the review (i.e., title and abstract screening, full-text reading, and data extraction), and in instances of disagreement, a third reviewer provided the deciding judgment.

Studies were searched on PubMed, Scopus, and Web of Science (updated September 2024) using terms related to NPs (e.g., nanoplastics, NPs), their effects (e.g., genotoxicity, cytotoxicity, hazard, damage), and study type (animal model, in vitro, cell line) combined with the Boolean operators “AND” or “OR”. The specific search string used for each database is available in Supplementary Materials—Table S1. Manual searches of the reference lists of the included studies and general search engines (e.g., Google) were also performed.

Studies were included if they met the PEO acronym, as follows: P—cell lines (animal or human); E—exposed to NPs (plastic particles below 1 µm); O—studies addressing the effects on cellular parameters (e.g., genotoxicity, cytotoxicity, hazard, damage, viability). Selection was restricted to English-language articles. Human studies, observational studies, reviews/commentaries), studies focusing on microplastics or sizes of particles not disclosed, those limited to co-exposure, and those without available data for extraction (no toxicity analysis or measurement of damage/harm) were excluded.

This systematic review was not registered in any protocol registry.

### 2.2. Data Extraction and Quality Assessment

Data extraction was performed using a pre-formulated spreadsheet in Excel^®^ with the following information: author names, year of publication, country, cell line, NP polymer type and size, concentration, time of exposure, methodology/effects evaluated, main findings/results, and DOI. The main findings included primary outcomes related to genotoxicity and cytotoxicity, which were measured through parameters such as cell viability, oxidative stress, inflammation, DNA damage, apoptosis, and cytoplasmic membrane damage. Secondary outcomes, which also pertain to cytotoxicity and cellular damage, included measures of cell proliferation, barrier integrity, lysosomal damage, autophagy dysfunction, ATP levels, and other bioenergetic parameters.

The methodological reporting quality of the selected studies was assessed using a checklist by Chierrito et al. (2019) [57] and visually represented through the ROBVIS tool [58]. For visual representation and to enhance interpretability, each parameter of the checklist was rated as “reported”, “not reported”, and “unclear reporting” or “not applicable”, which was translated into “low”, “high”, and “unclear” risk of bias or no information, respectively.

### 2.3. Data Visualization

Descriptive statistics are used to report the findings. A violin plot was generated using Microsoft Power BI to illustrate data distribution. Heatmaps were constructed in Microsoft Excel through conditional formatting, employing a gradient of dark teal (Accent 1) for 0%, white for 50%, and dark red for 100%. Percentages were calculated as the ratio of reported negative effects to total reported effects. The conditional formatting colors were subsequently replicated in Microsoft PowerPoint to develop a corresponding scale, designed with 25% incremental size increases for improved visual distinction. Additional graphics were created using Excel’s built-in visualization tools.

## 3. Results

### 3.1. Overall Results

**Figure 2:**
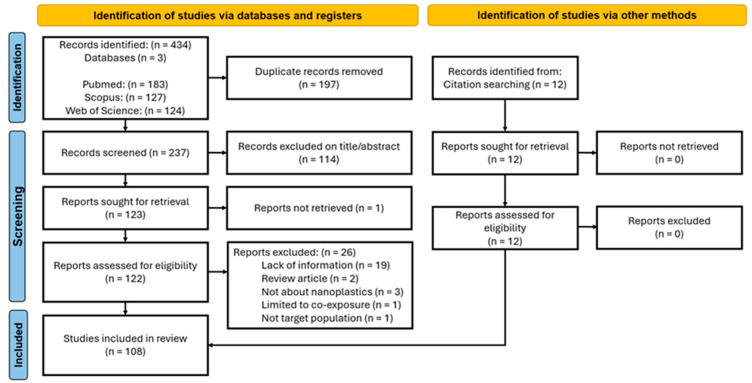
Flow diagram of the systematic review process.

The initial search yielded a total of 434 articles from various databases. After removing duplicates, 237 unique were screened (title/abstract reading), of which 114 were considered irrelevant for the research. From the 122 studies assessed for eligibility (full-text reading), 26 were excluded. A total of 12 articles were additionally found during manual searching, finally resulting in 108 studies for synthesis [34,35,46,47,48,59,60,61,62,63,64,65,66,67,68,69,70,71,72,73,74,75,76,77,78,79,80,81,82,83,84,85,86,87,88,89,90,91,92,93,94,95,96,97,98,99,100,101,102,103,104,105,106,107,108,109,110,111,112,113,114,115,116,117,118,119,120,121,122,123,124,125,126,127,128,129,130,131,132,133,134,135,136,137,138,139,140,141,142,143,144,145,146,147,148,149,150,151,152,153,154,155,156,157,158,159,160,161,162] (Figure 2).

**Figure 3:**
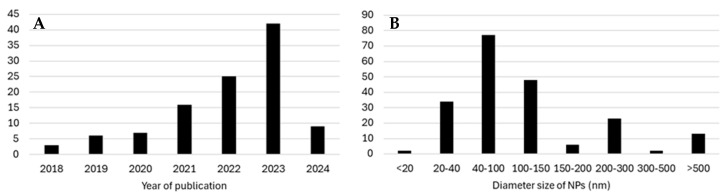
Study characteristics according to: (**A**) distribution of articles by year of publication; (**B**) distribution of articles by NP size categories reported.

The studies were conducted in 20 different countries, with the majority being in China (n = 58; 54%), followed by Spain and Italy (14% and 9%, respectively). The studies were published between 2018 and 2024, with a higher prevalence (85%) in the last 4 years (see Figure 3).

**Figure 4:**
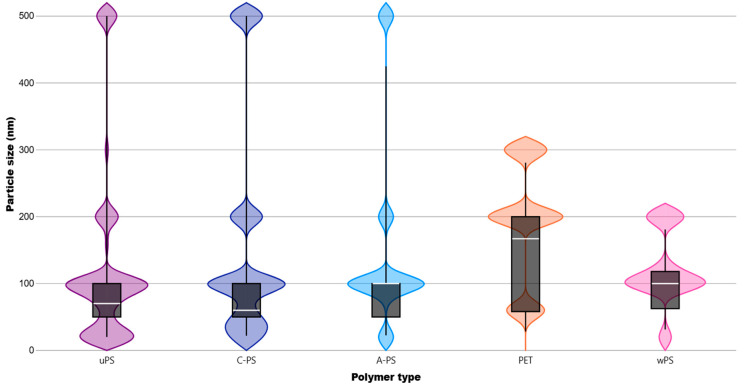
Median value distribution of polymer by the most used NP polymers. uPS = unmodified polystyrene. C−PS = carboxyl-modified polystyrene. A−PS = amine-modified polystyrene. PET = polyethylene terephthalate. wPS = weathered polystyrene.

The main NP polymers mentioned in the studies were PS, primarily unmodified or pristine PS (uPS; 58%), but also carboxyl-modified (C-PS; 14%), amino-modified PS (A-PS; 13%), and polyethylene terephthalate (PET), with a much lower representation of 3%. In total, PS-NPs, including all modifications and weathering processes, accounted for 90% of NPs described in these studies. The average NP size in solution varied between 15 nm and approximately 531 nm, with a greater prevalence fitting into the 40–100 nm category (38%), followed by 100–150 and 20–40 nm (24% and 17%, respectively) (see Figure 2). A violin plot (Figure 4) better illustrates the distribution of sizes by most used NP polymer types.

Table 1 summarizes the negative effects on cellular parameters (e.g., genotoxicity, cytotoxicity, hazard, damage, cell viability) caused by the main types of NPs in each organic system. The main findings of the included studies are addressed in the sections below. Complete information is available in Supplementary Materials (Tables S2–S10).

NPs have been shown to accumulate and exert toxic effects in various organs, depending on the physicochemical properties of the particles, the route of exposure, and the administered dose. Overall, respiratory cell lines were the most affected by NP exposure (65–100% of studies). Nervous, urinary, and connective tissue systems were also highly vulnerable (80–100% of reported cases). In contrast, hepatic, digestive and immune systems were more resilient, with damage reported in 60–70% of studies.

### 3.2. Results by NP Polymer Type and Size

**Figure 5:**
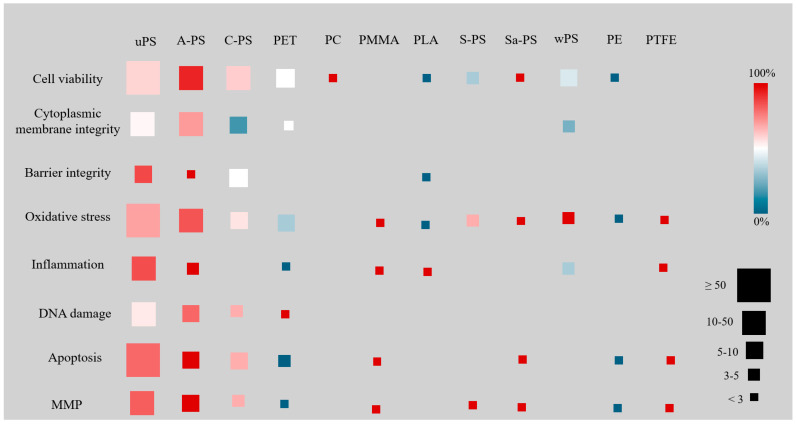
Toxic effects caused by NPs on eight different parameters according to polymer type. uPS = unmodified polystyrene, A−PS = amine-modified polystyrene, C−PS = carboxyl-modified polystyrene, PET = polyethylene terephthalate, PC = polycarbonate, PMMA = polymethyl methacrylate, PLA = polylactic acid, S-PS = sulfate-modified polystyrene, Sa−PS = sulfonic acid-modified polystyrene, wPS = weathered polystyrene, PE = polyethylene, PTFE = polytetrafluoroethylene.

**Figure 6:**
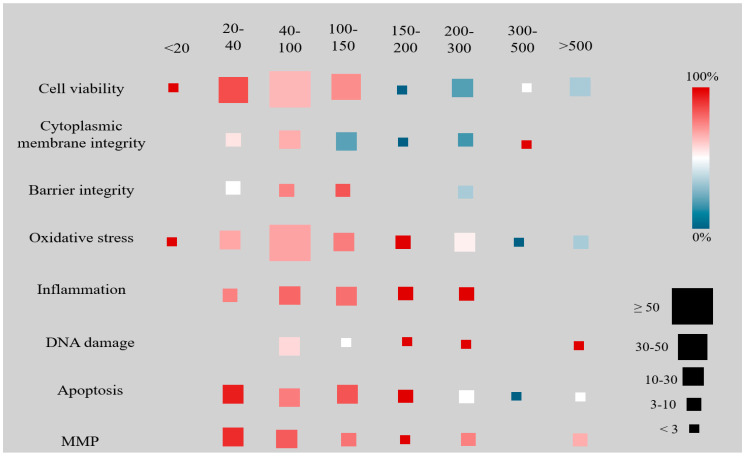
Toxic effects caused by NPs on eight different parameters according to size category (range from <20 to >500 nm).

Heatmaps were generated to depict the relationships between toxicity and polymer types (Figure 5), as well as toxicity according to size categories (Figure 6).

Among NP polymers, both uPS and A-PS showed higher toxicity, with negative effects reported in over 50% of the studies for all main cellular parameters (cell viability, alterations on cytoplasmic membrane integrity or barrier integrity, oxidative stress, inflammation, DNA damage, apoptosis, matrix metalloproteinases—MMPs). Moreover, A-PS appeared more toxic than uPS, a finding confirmed by studies using particles of the same size. C-PS showed some toxicity, but had less impact on membrane and barrier integrity, while no data were reported on inflammation. Although PET caused negative effects in less than 50% of studies. One report associated the exposure to these NPs with significant DNA damage. Few studies focused on wPS, yet it was related to increased oxidative stress. Polymers such as PC, Sa-PS, and PTFE were consistently associated with negative outcomes, while PE and PLA caused less damage. PMMA and S-PS showed mixed results, though these were based on limited data, often from single studies.

From a broader view, NPs measuring up to 150 nm consistently caused more cellular damage across most assessed parameters. In contrast, particles larger than 150 nm showed more variable results: while effects on cell viability and membrane integrity were less frequent, oxidative stress, inflammation, DNA damage, apoptosis, and MMP remained common up to 300 nm. The largest NP categories were less frequently studied and generally showed fewer toxic effects.

### 3.3. Results by Biological System

Twelve studies [59,84,99,104,105,109,119,120,131,138,151,156] investigated the effects of eleven different NP polymers (uPS, C-PS, A-PS, S-PS, wPS, PET, PC, PMMA, PLA, and PP) on seven different cell lines belonging to the hepatic system: LO2, HepG2, second-generation UHHs, HepaRG, ZFL, rat hepatocyte suspensions, and AML-12. A reduction in cell viability was observed in 45% of studies with uPS exposure, especially with particles <100 nm. Membrane integrity, inflammation, and mitochondrial membrane potential were negatively affected in 50% of the studies, while around 63% of reports also mentioned oxidative stress. DNA damage and increased apoptosis were less frequent. Similarly, C-PS (n = 3/4 studies; 75%) and A-PS 50 and 100 nm (n = 2/2; 100%) reduced cell viability. wPS, tested in one study, caused decreased viability, increased oxidative stress, and reduced inflammatory response. PET also reduced cell viability in all three tested sizes (58–252 nm). PC reduced viability in two studies (31.5–47 nm), with one of them additionally reporting impaired membrane integrity, reduced albumin production, and disrupted cytochrome P450 function. PMMA and PLA showed minimal effects, mostly inflammation. PP (158 nm) and S-PS (100 nm) had no significant toxicity in this system.

Only four studies [76,90,106,131] evaluated the effects of uPS and wPS on two human renal cell lines: HK2 and 293T. Most nanoparticles were between 20 and 150 nm (n = 7/9; 78%), with larger particles (150–500 nm) showing no effect on viability. uPS 20 and 60 nm exhibited the highest toxicity based on cell viability. Both uPS and wPS presented toxic effects associated with a decrease in cell viability, cytoplasmic membrane integrity, barrier integrity, and MMP, as well as an increase in oxidative stress, inflammation, and apoptosis.

A total of 15 studies [63,69,74,78,86,88,92,107,121,127,136,140,145,153,158] investigated the effects of four different NP polymers (uPS, A-PS, C-PS, PET) on seven respiratory cell lines, including human (A549, BEAS-2B, HPAEpiC, HNEpCs), murine (MLE-12, MH-S), and fish (RTgill-W1). The most frequently studied cell line was A549 (n = 7), followed by BEAS-2B (n = 5). uPS exposure led to higher negative effects in respiratory cell lines compared to other organic systems, with studies consistently reporting decreased cell viability and increased levels of oxidative stress, inflammation, and apoptosis whenever these parameters were evaluated. Some studies also reported mitochondrial dysfunction, disrupted energy metabolism, elevated endoplasmic reticulum stress, autophagy, and S-phase cell cycle arrest as consequences of uPS exposure. A-PS exposure yielded similar results, albeit fewer studies examined this polymer. C-PS NPs also impacted cell viability consistently, while oxidative stress and DNA damage were present in 50% of the reports. Membrane integrity was not affected by this polymer, although only one report was available. PET NPs, studied in A549 cell lines, impacted oxidative stress (n = 2/2; 100%), DNA damage (n = 1/1; 100%), and cell viability (n = 1/2; 50%), while single reports for membrane integrity, MMP, and apoptosis found no significant effects. Mitochondrial impairment was linked with smaller particles (uPS 20, uPS 50, and A-PS 20 nm). The accumulation of Fe^2+^ was proposed by one study as a contributor to the toxicity observed from uPS exposure.

The effects of NPs on gastric and intestinal cell lines were evaluated by 33 articles [34,46,62,66,67,70,75,82,87,89,91,92,94,96,101,102,111,115,119,122,126,129,134,135,137,138,141,149,150,152,154,157,162]. Overall, 17 cell lines, including human lines such as Caco-2, GES-1, HT29 and HT29-MTX-12, HCT-116, HIEC6, RKO, NCM460, HET-1A, HEEC, SNU-1, murine IEC-6, and fish RTgutGC were assessed. Two intestinal organoids (from mouse and human HiPSCs), four co-cultures involving Caco-2 and HT29 (with or without modifications), and two tricultures (Caco-2/HT29 with THP-1 or M-cells) were also studied. Overall, uPS showed negative effects on cell viability (61% of studies), oxidative stress (68%), and mitochondrial membrane potential (73%). Apoptosis was elevated in 86% of studies, while barrier integrity and inflammation were less frequently affected (75% and 63%, respectively). Specific cell lines like GES-1 and SNU-1 showed reduced viability and proliferation, and oxidative stress increased with particles up to 500 nm. Smaller particles (50–60 nm) had a stronger impact on apoptosis and membrane potential. C-PS NPs were also found to impact cell viability (43% of studies), barrier integrity (50%), and oxidative stress (33%). A-PS was also found to affect cell viability (71%), cytoplasmic membrane integrity (75%), and barrier integrity (50%). DNA damage and apoptosis were significantly increased in all cases exposed to this particle. PET NPs, studied in Caco-2 monocultures, showed variable results depending on size. Smaller nanoparticles (26.7–30 nm) had no toxicity, while larger ones (58–252 nm) reduced cell viability and influenced DNA damage and membrane integrity. One study suggested a shift toward anaerobic conditions with weathered PET, while pristine PET promoted aerobic energy production. A decrease in cell viability was reported with PET 58–252 nm, but oxidative stress and cell cycle distribution were unaffected. Other NPs (PLA, PMMA, PTFE, PP, PC) presented fewer effects on the gastrointestinal system.

A total of 22 studies [47,60,61,64,65,75,77,79,81,103,108,112,117,118,124,128,143,148,154,157,159,161] investigated the effects of three NP polymers (PS, PE, and PET) on 11 immune cell lines from mice, fish, sea urchins, and humans. The most frequently studied cell line was the murine RAW264.7 (n = 8), followed by human THP-1 (n = 6), Raji-B (n = 3), and TK6 (n = 3). uPS exposure led to fewer negative effects in these cell lines compared to other organic systems, with 38% of studies showing reduced cell viability, 46% oxidative stress, and 50% cytoplasmic membrane integrity and DNA damage, yet inflammation, apoptosis, and MMP disruption were reported by 75%, 83%, and 77% of studies, respectively. No data were available for barrier integrity. A-PS and C-PS also negatively affected all cell lines in cell viability, membrane integrity, apoptosis, and MMP, yet in lower proportions (<50% of studies). S-PS, evaluated in RAW264.7 and THP-1 cells, reduced cell viability at concentrations ≥ 250 µg/mL, decreased MMP, and increased oxidative stress and lipid content. Sa-PS20 caused toxicity in mice splenocytes at 40 µg/mL, affecting cell viability, oxidative stress, apoptosis, and MMP. PE increased inflammation in RAW264.7 cells, elevating IL-1β production and CD80 expression, while PET had no significant effects on cell viability, oxidative stress, or apoptosis. Phagocytosis, crucial for immune cells like macrophages, neutrophils, and dendritic cells, was impaired by most NP exposure, as indicated by lysosomal damage and altered phagocytic capacity.

Different reproductive system cells (including murine TM3, TM4, GC2, swine granulosa cells, oyster oocytes and spermatozoa, and human cell lines KGN, HeLa, HEY, and NTERA-2) were evaluated by 13 studies [68,73,93,97,98,100,106,113,116,130,132,146,161]. Around 70% of studies reported decreased cell viability upon uPS exposure. Other negative effects included impaired cytoplasmic membrane integrity, barrier integrity, inflammation, apoptosis, and MMP. A-PS exposure also negatively affected cell viability (increased mortality) in oyster oocytes and increased oxidative stress in human spermatozoa (n = 1 study). Human spermatozoa also showed DNA damage and reduced membrane integrity when exposed to A-PS50, but no significant effects were seen with A-PS100. Moreover, hormonal imbalances and damage to other organelles such as the acrosome were observed with exposure to uPS and A-PS. C-PS reduced both cell viability and elevated oxidative stress in oyster oocytes. Three studies (23%) additionally identified oncogenic potential from NP exposure linked to the activation of several metabolic pathways, including MAPK, PI3K-AKT, and Hippo signaling. Some authors (n = 4 studies) [59,72,142,150] also assessed the impact of NP on gestational tissue by means of human-origin cell lines BeWo b30 (n = 2), HTR8/SVneo (n = 1), and HUVEC (n = 1). PS polymers were the only ones studied. While uPS and wPS did not cause significant changes in cell viability, A-PS and C-PS led to decreases in this parameter. A-PS additionally affected membrane integrity, oxidative stress, and MMP and altered mitochondrial gene expression. Despite limited toxicity evidence, uPS increased oxidative stress, inflammation, apoptosis, and decreased MMP. Specific exposure to uPS100 in HTR8/SVneo cells reduced trophoblast migration and invasion and caused 344 differentially expressed genes, with 87% (298 genes) being downregulated.

Another 12 studies [71,80,83,85,95,110,114,123,125,139,155,160] involving 10 different cell lines (murine HT22, BV2, human hCMED/D3, murine C17.2 and primary hippocampal NSCs, fish cell lines DLB-1 and Fub-1, and human SH-SY5Y and hNS1) assessed the nervous system. Again, uPS negatively affected cell viability in 78% of studies and induced apoptosis in 33%. It also caused negative effects on barrier integrity, oxidative stress, inflammation, and MMP. Other observed effects included increased autophagy, mitochondrial dysfunction, changes in nuclear staining, and the activation of the GAPDH/Ac-Tau signaling pathway, linked to neurodegenerative diseases. One study found alterations in gene expression related to oxidative stress, DNA repair, inflammation, and apoptosis in the hNS1 cell line, indicating a multifaceted cellular response to uPS exposure. Similarly, A-PS affected cell viability, barrier integrity, oxidative stress, inflammation, and apoptosis (in around 50% of reports). Conversely, C-PS and PE did not show significant cellular toxicity (less than 30% of studies). In primary hippocampal NSCs exposed to C-PS NPs (50 and 500 nm), cell proliferation, number of cells, cell diameter, and neuron length were slightly reduced.

Finally, six studies [48,110,124,133,144,147] on connective tissue cell lines (human Hs27, swine ASCs, murine MC3T3-E1 and MLOY-4, and fish ZF4 and SAF-1) were included. uPS negatively impacted cell viability in 75% of cases and increased oxidative stress in 83%. It also increased inflammation, DNA damage, and apoptosis. Specifically, uPS50 reduced the migratory ability of MC3T3-E1 cells, decreased bone deposition, and increased bone resorption. Swine ASCs showed reduced cell viability (yet with no affected cell proliferation), while Hs27 cells experienced decreased cell proliferation when exposed to uPS100 at similar concentrations. Exposure to uPS94 in the H9C2 cell line increased autophagy and activated the TGF-β1/Smad signaling pathway, which is associated with fibrosis.

### 3.4. Reporting Quality of the Studies

**Figure 7:**
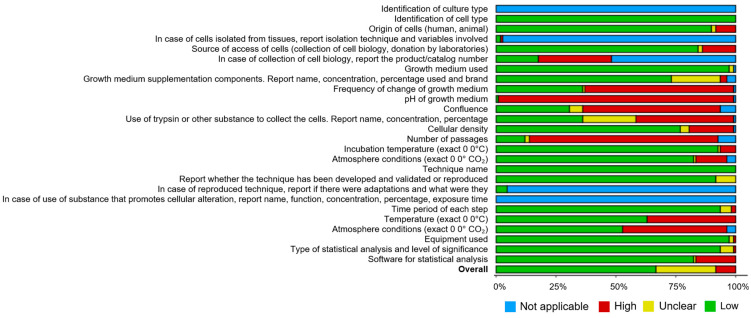
Summary plot of risk of bias assessed according to Chierrito et al. (2019) [57].

The results of the risk-of-bias assessment are summarized in Figure 7. Overall, studies demonstrated low to moderate risk of bias (60%). The most common concerns were related to the lack of information on: pH of growth medium (only one study reported this), number of passages (about 12% of studies gave this information), frequency of chpalaange of growth medium/confluence (less than 40% mentioned this variable) and substance used for cell collection (36% presenting low risk of bias).

## 4. Discussion

This systematic scoping review is the first to synthesize evidence on the negative effects of exposure to different NP types on cellular parameters across 108 in vitro studies, highlighting the primary biological systems affected. Although microscopic plastic debris was first reported in 2004 [163], public and scientific attention to NPs surged following a widely publicized 2019 [164] study on their release from plastic teabags, correlating with a sharp rise in research on the topic (we found over 80% of studies published in the last five years), particularly driven by Asian countries. In fact, since the mid-1990s, China has experienced an important increase in scientific output, becoming one of the top publishers in 2022 [165,166,167], a trend that coincides with its status as the largest generator of unmanaged plastic waste, though its per capita figures remain modest due to its large population [168].

In our review, we found a clear trend in NP polymer selection, with almost 90% corresponding to PS (including surface modifications and weathering processes), often represented by spherical nanobeads. This trend may be linked to the ease of synthesizing these polymers at the nanoscale [5,169]. However, these findings may not accurately reflect environmental NPs, as PE is the most prevalent polymer, both in production and abundance in marine environments, accounting for 30% and 23%, respectively [170,171]. PP follows as another major polymer, with approximately 20% representation [172]. Regarding size, most NPs had diameters of up to 150 nm, with medians below 100 nm for the most used PS NPs. This may reflect the lack of consensus on the definition of NPs, with some researchers considering particles with diameters of 100 nm or less as NPs [7,173]. In this context, further studies should focus on processing NP samples (especially PE) to closely mimic their natural state, as they would exist in biological models. This includes subjecting the particles to chemical or physical processes that replicate biological conditions (such as digestion), ensuring more accurate and realistic exposure scenarios. Moreover, fibers and fragments should be tested as they also present toxicity risks.

Overall, we found that exposure to A-PS and uPS was associated with the highest levels of cellular damage, impairing cell viability, cytoplasmic membrane integrity, and inducing DNA damage. Parameters such as inflammation, MMP, and apoptosis were also frequently affected by these polymers. C-PS also negatively affected different cell lines, though to a lesser extent. Conversely, polymers such as Sa-PS, PC, PE, and PTFE consistently showed fewer negative effects, while wPS, S-PS, and PMMA yielded mixed results, although data on these polymers were very limited. The toxicity and environmental impact of polymeric nanoparticles depend on several factors, including size, morphology, hydrophobicity, surface area, surface charge, and composition [174,175,176,177]. Surface functional groups, such as carboxyl and amine groups, introduce surface charges that influence particle–protein interactions and interactions with the cytoplasmic membrane, potentially affecting uptake and toxicity. Due to the predominantly negative charge of the cytoplasmic membrane, positively charged particles like A-PS are more readily absorbed than negatively charged ones like C-PS, contributing to the higher toxicity observed in A-PS [178]. This phenomenon has been documented in some studies and may help explain the higher frequency of toxicity observed with these particles. The zeta potential, which measures the electrical potential of particles in suspension, is also an important parameter to consider in nanotoxicity studies due to its relationship with surface charge [105,179].

PET nanoparticles had no effect on inflammation, apoptosis, or MMP, with limited negative impacts on other cellular parameters. PET NPs under different conditions exhibit negative zeta potentials of distinct magnitudes [180], which on their own could be related to the lower toxicity associated with this polymer. Moreover, PET used in the included studies were larger on average compared to PS NPs. The toxicity of NPs has been shown to be size-dependent, with smaller particles typically inducing more severe effects or doing so at lower concentrations than larger particles [34,60]. However, the contradictory results observed for similar particles in some studies suggest that factors such as cell susceptibility, polymer type, concentration, and other previously mentioned variables significantly influence cellular responses. Moreover, the variability in particle size distribution across studies complicates the accurate interpretation of results, highlighting the need for further statistical analyses.

The literature shows that NPs induce cytotoxicity and genotoxicity through several mechanisms. A primary mechanism is oxidative stress, where NPs generate reactive oxygen species (ROS), leading to cellular damage and apoptosis [11,181,182]. Inflammation is another significant pathway, as NPs can trigger the release of pro-inflammatory cytokines [11,182]. Direct cellular interactions, such as membrane disruption and interference with signaling pathways, also contribute to NP-induced toxicity. Regardless of the mechanism, cell viability is a key parameter used to determine the concentration at which a substance begins to exhibit toxicity, as indicated by EC50 and LD50 values [183]. In our review, most studies focused on acute exposure, leaving a gap in understanding the long-term effects of chronic NP exposure and their mechanisms of action. Assessing the persistence and cumulative toxicity of NPs may be challenging with cell lines due to limitations in passage numbers and the potential genetic and phenotypic changes that immortalized cells may undergo over time.

Our findings suggest that respiratory cell lines such as A549 are particularly susceptible to NP exposure, followed by cell lines from the nervous, urinary, and connective tissues systems. On the other hand, the hepatic, digestive, and immune systems had the lowest frequency of negative effects from NP exposure, which may be attributed to the detoxification, transformation, and defense roles of these systems, respectively. In fact, hepatocytes, specialized in metabolizing and clearing toxins, use enzymes like cytochrome P450 to neutralize harmful substances [184,185,186,187,188,189,190]. Yet, while key cellular parameters often had no significant effects from NP exposure, some hepatotoxic outcomes, such as mitochondrial damage and necroptosis in macrophages leading to hepatocyte injury, are only detectable in more complex liver microenvironments [184,185,186,187,188,189,190]. NP exposure has also been linked to disruptions in lipid and energy metabolism (ATP/ADP/AMP), affecting processes like glycolysis and fatty acid synthesis [190,191,192]. Additionally, the overproduction of ROS, identified as a key event in NP-mediated digestive injury, along with effects such as oxidative stress, inflammation, and metabolic disorders, were consistently reported in some studies reviewed. These effects should be further explored, particularly in relation to the role of gut microbiota in disease development [193,194,195]. This is especially important considering that NPs can translocate through the digestive epithelium and also accumulate in the kidneys. During digestion, NPs may aggregate or change size, affecting their toxicity. Research has shown that PS NPs can cause nephrotoxicity, inflammation, oxidative stress, and biomarker alterations. However, more studies are needed to understand how aggregation and protein corona formation influence NP behavior and kidney toxicity [196,197,198]. Moreover, a comprehensive understanding of NP-induced toxicities could be achieved by combining adverse outcome pathways (AOPs) with advanced models such as organoids and organ-on-chip systems, which more accurately replicate biological functions in vitro [199,200].

Similarly, to the hepatic and digestive systems, immune cells, including macrophages, neutrophils, and lymphocytes, play a key role in defending the body against foreign contaminants through phagocytosis and various immune responses to neutralize and eliminate threats. These protective mechanisms may contribute to the observed resilience to NP-induced toxicity, reducing the frequency and severity of adverse effects compared to other cell types [201]. However, some studies, particularly those using THP-1 macrophage cell lines derived from peripheral blood of leukemia patients, report reduced cell viability, damage to the cytoplasmic membrane, and increased necrosis following exposure to 500 nm PS beads [202,203]. Although variability among leukocyte cell lines complicates the understanding of NP effects, Wolf et al. (2023) [204] emphasized that smaller particles tend to have greater toxicity, with macrophages being the most sensitive and T cells the most resistant. Additionally, exposure to plastic particles affected immune checkpoint marker expression in all immune cell subpopulations tested and induced an M2 phenotype in macrophages, suggesting a downregulation of the inflammatory M1 phenotype.

The respiratory system may face more significant risks from NPs compared to other organs due to their ability to pass through the airway and interact with cells. NPs, particularly small ones, can easily penetrate the alveolar membrane, reaching the lungs and potentially causing both acute and chronic damage. Moreover, during their transit through the respiratory tract, NPs may acquire a pulmonary surfactant corona, which alters their surface properties and subsequently influences their interaction with lung cells. This modification could affect their cellular uptake, toxicity, and overall risk profile. For instance, surfactant proteins can change the surface charge and hydrophobicity of the particles, potentially increasing the inflammatory response and cellular damage [205,206]. Further in vitro studies using more sophisticated lung models, such as co-cultures of epithelial and immune cells, are needed to better replicate the complex lung environment. These models could help assess the inflammatory response, oxidative stress, and potential fibrosis development following NP exposure. Additionally, in vivo studies using animal models, particularly those that simulate real-life exposure scenarios (e.g., repeated low-level exposure), are essential to determine the long-term effects of NP accumulation in the lungs and the overall pulmonary health risks [205,206].

For the nervous system, while some NPs may cross the blood–brain barrier, their penetration is generally limited (2–6%), especially for particles larger than 10 nm [207]. However, some studies suggest that PS NPs could cross the BBB and induce neurotoxicity (by means of interaction with microglial cells, neurons, and endothelial cells, triggering inflammatory responses, oxidative stress, and neuronal apoptosis), warranting further investigation in vivo [125,208]. Further in vitro studies using brain models, such as brain organoids or co-cultures of neurons and glial cells, should be employed, as they can more closely mimic the interactions between NPs and different cell types in the brain.

Similarly, to the nervous system, while NPs are unlikely to penetrate connective tissues of the stratum corneum (usually particles smaller than 100 nm), this ability can be significantly influenced by the condition of the skin [209,210], which raises concerns about their ability to affect underlying tissues such as muscle, bone, adipose tissue, and fibroblasts. Atopic dermatitis models show increased NP accumulation in inflamed skin, also highlighting the importance of vehicle solutions in NP absorption. For example, when combined with solvents like dimethyl sulfoxide or ethanol, NPs of various sizes are able to penetrate deeper into the skin, sometimes reaching the stratum granulosum and deeper layers of the epidermis and potentially damaging them through inflammation, oxidative stress, or cellular apoptosis [211,212,213,214]. Advanced skin models such as 3D skin cultures could provide more accurate insights into the behavior of NPs in these environments.

Although fewer studies have focused on the reproductive organs, existing evidence suggests that NPs can accumulate and exert toxic effects on both male and female systems. Animal studies have shown that oxidative stress and inflammation contribute to impaired sperm vitality and endocrine disruption, along with hormonal imbalances, oncogenic metabolic pathway activation, and apoptosis [215,216]. In gestational tissues, limited human cell line studies indicate that NP exposure can negatively affect cell viability, oxidative stress, inflammation, and membrane integrity, potentially impacting fetal development and organ function [217,218,219]. To better understand these effects, further well-designed in vitro and in vivo studies are needed.

In fact, most studies in this review exhibited a moderate risk of bias, primarily due to insufficient reporting of key factors such as medium pH, medium-changing frequency, and passage number. Standardizing methodologies and reporting protocols is essential to enhance both reproducibility and comparability across studies. Furthermore, researchers need to reach a consensus on NP exposure protocols, including parameters like size ranges, concentrations, surface area, and particle numbers, with well-justified reasoning. Particle properties such as buoyancy and adaptation to dynamic exposure models should also be reported. Discrepancies in these factors make cross-study comparisons challenging and hinder the ability to draw definitive conclusions. For example, the same concentration of smaller particles results in a higher surface area and particle number, which could lead to more pronounced biological effects.

Our study has some limitations. Being a systematic review, the focus was primarily descriptive, and no statistical analyses were performed. This approach was chosen to map the preliminary landscape of this underexplored topic, and while international guidelines for conducting and reporting were followed, our findings may serve as a foundation for future research. Although the scoping review methodology does not require a detailed risk-of-bias assessment, to provide insight into the reporting quality of the included studies, we adapted a checklist tailored to in vitro cellular models. Other tools may lead to slightly different results. The lack of standardized methodologies and reporting, resulting in high heterogeneity across studies, may limit the generalizability and reproducibility of some findings. Despite these challenges, the included studies represent the best available evidence on the topic. Lastly, while various cellular damage parameters, such as oxidative stress, apoptosis, and inflammation, were assessed, the molecular mechanisms driving NP toxicity require further investigation.

## 5. Conclusions

This systematic review highlights the complex and multifaceted nature of NP toxicity, demonstrating that factors such as size, surface charge, and polymer type significantly influence the harmful effects of these particles—especially on the respiratory system. Over the last decade, there has been an exponential growth in research on NP toxicity, particularly in countries like China, which has become a leading force in studies in this field. Our findings indicate that a polymer’s physical and chemical properties play a crucial role, with PET showing lower toxicity potential compared to PS. However, the current research predominantly focuses on spherical PS particles, underscoring the need for more studies on other nanosized polymers to form comprehensive conclusions. The surface charge of NPs also emerged as a critical factor, with positively charged A-PS particles exhibiting higher toxicity across various cell lines, while negatively charged particles like C-PS were less toxic. Respiratory cell lines, particularly A549 and BEAS-2B, were more susceptible to NP exposure, in contrast to hepatic and immune system cell lines, which showed greater resilience likely due to their biological detoxification and defense functions. Digestive cell lines had multiple responses, with cancer cell lines demonstrating higher resistance compared to healthy ones, as expected. This review identifies significant knowledge gaps, particularly the reliance on high concentrations of spherical PS particles, which do not accurately reflect real environmental conditions. Also, the lack of standardized methodologies further complicates the comparability of studies, as the same parameter may be obtained through different methods.

To address these issues, future research should prioritize the use of diverse NP types and shapes, standardized methodologies, environmentally relevant exposure levels, and biologically relevant acute and chronic models to improve the accuracy and ecological validity of NP toxicity assessments. Advanced in vitro models like organoids and organ-on-chip systems that better replicate the physiological conditions of tissues and organs, as well as using environmentally relevant plastic particles and processing of NPs to mimic their natural state, are essential to better simulate physiological conditions and provide more accurate toxicity assessments. These steps are essential to fill the existing knowledge gaps and accurately determine the environmental and health impacts of NPs.

## Figures and Tables

**Table 1:** Main findings from the included studies regarding to the negative effects on cellular parameters caused by the main types of NPs per biological system.

System	N. Articles	N. Cellular Lines	NP Types	Global Negative Effects of the Most Reported NPs (% of Studies) #		
				C-PS	uPS	A-PS
Hepatic	12	7	uPS, C-PS, A-PS, wPS, PET, wPET, PC, PMMA, PLA, PP, S-PS	57	42	100 *
Urinary	4	2	uPS, wPS	-	85	-
Respiratory	15	7	uPS, C-PS, A-PS, PET	64	100	100
Digestive	33	13	uPS, C-PS, A-PS, wPS, PET, wPET, PC, PMMA, PLA, PP, PTFE	41	62	75
Immune	22	11	uPS, C-PS, A-PS, S-PS, Sa-PS, PE, PET	78	52	85
Reproductive	13	11	uPS, C-PS, A-PS	100 *	80	62.5
Gestational tissues	4	3	uPS, wPS, A-PS, C-PS	33	40 *	100 *
Nervous	12	10	uPS, C-PS, A-PS, PE	0	80	100 *
Connecting tissues	6	7	uPS, A-PS	-	87 *	100 *

# Negative effects included reduced cell viability, alterations on cytoplasmic membrane integrity or barrier integrity, enhanced oxidative stress, inflammation, DNA damage, and apoptosis or alteration in matrix metalloproteinases (MMPs). * Few studies (<5). A−PS = amine-modified polystyrene, C−PS = carboxyl-modified polystyrene, PC = polycarbonate, PE = polyethylene, PET = polyethylene terephthalate, PLA = polylactic acid, PMMA = polymethyl methacrylate, PP = polypropylene, PTFE = polytetrafluoroethylene, S−PS = sulfate-modified polystyrene, Sa−PS = sulfonic acid-modified polystyrene, uPS = unmodified polystyrene, wPET = weathered polyethylene terephthalate, wPS = weathered polystyrene.

## Data Availability

No new data were created or analyzed in this study.

## Supplementary Materials

The following supporting information can be downloaded at https://www.mdpi.com/article/10.3390/jox15030075/s1. Table S1: Search strategies; Table S2: Summary of hepatic cell line studies; Table S3: Summary of urinary cell line studies; Table S4: Summary of respiratory cell line studies; Table S5: Summary of digestive system cell line studies; Table S6: Summary of immune system cell line studies; Table S7: Summary of reproductive system cell line studies; Table S8: Summary of gestational tissue cell line studies; Table S9: Summary of nervous system cell line studies; Table S10: Summary of connective tissue cell line studies.

## Abbreviations

The following abbreviations are used in this manuscript:

AOP	adverse outcome pathway
A-PS	amine-modified polystyrene
C-PS	carboxyl-modified polystyrene
ECHA	European Chemicals Agency
MMP	mitochondrial membrane potential
NP	nanoplastics
PET	polyethylene terephthalate
PE	polyethylene
PC	polycarbonate
PLA	polylactic acid
PMMA	polymethyl methacrylate
PP	polypropylene
PS	polystyrene
PTFE	polytetrafluoroethylene
ROS	reactive oxygen species
Sa-PS	sulfonic acid-modified polystyrene
S-PS	sulfate-modified polystyrene
uPS	unmodified polystyrene
wPET	weathered polyethylene terephthalate
wPS	weathered polystyrene
