# Supplementary material for: Assessing the Impact of Nanoplastics in Biological Systems: Systematic Review of In Vitro Animal Studies
Source: J Xenobiot. 2025 May 17;15(3):75. doi: 10.3390/jox15030075 (PMC12101406; doi:10.3390/jox15030075)
Supplement: Supplementary file 1 [file jox-15-00075-s001.zip › jox-3620790-supplementary.pdf]

Supplementary Material

1. Search strategies

**PubMed**	(nanoplastics[TIAB] OR NPs[TIAB]) AND (damage[TIAB] OR effects[TIAB] OR hazard[TIAB] OR toxicological[TIAB] OR toxicity[TIAB] OR genotoxicity[TIAB] OR cytotoxicity[TIAB]) AND (“Cell line”[MH] OR “cell line”[TIAB] OR “cellular line”[TIAB] OR “cell culture”[TIAB] OR “in vitro”[TIAB])
**Scopus**	TITLE (nanoplastics OR NPs) AND TITLE-ABS (damage OR hazard OR effects OR toxicity OR toxicological OR genotoxicity OR cytotoxicity) AND TITLE-ABS ("cell line" OR "cellular line" OR "cell culture" OR "in vitro")
**Web of Science**	((TI=(nanoplastics OR NPs)) AND TS=(damage OR effects OR hazard OR toxicological OR toxicity OR genotoxicity OR cytotoxicity)) AND TS=("cell line" OR "cellular line" OR "cell culture" OR "in vitro")

2. Summary of hepatic cell line studies

Authors	Year	Country	Cellular line	Polymer type	NP size (nm)	NP concentration	Exposure duration	Main findings
**Brandts, I. et al.**	2020	Spain	ZFL	uPS	≈ 65	0.05, 0.5, **5**, 10, 25, **50**, 100, 250, 500 and 1000 mg/L	12, 20, 24h and 36h	↓: Cell viability (20h, >100 mg/L)<br>=: Antiviral response and cell peroxidation status
**Englert, F.H. et al.**	2023	Switzerland	HepaRG	uPS	25 and 100	0.1–1000 µg/mL (**50** µg/mL)	24h and 10 days	=: Cell viability (uPS25/100), oxidative stress (uPS25/100)<br>↑: Inflammation[#] and lipid accumulation[#] (uPS25/100)
				wPS				↓: Cell viability (wPS25/100)<br>↑: Oxidative stress (wPS25, >3.6 µg/mL; wPS100, >49.8 µg/mL)<br>=: Inflammation[#] (wPS25/100)
**He, Y. et al.**	2020	China	HepG2	uPS	50	**10, 50,** and **100** µg/mL	24h	↓: Cell viability[+] (≈75% with 100 µg/mL and ≈84% with 50 µg/mL)<br>Others: Study suggests increase in oxidative stress, but the statistical analysis is not well elucidated. A-PS and C-PS showed more severe cytotoxicity than uPS.
				C-PS				
				A-PS				
**Huang, J. et al.**	2023	China	AML-12	uPS	20, 50, 100, 200 and 500	10, 25, 50, 100, **200**, 400 and 800 µg/mL (**300** µg/mL)	4h	↓: Cell viability (uPS20, > 400 µg/mL) and MMP (uPS20, 200 µg/mL)<br>↑: Apoptosis (uPS20, 200 µg/mL) and oxidative stress (uPS20, 300 µg/mL)<br>=: MMP (uPS100), cell viability, oxidative stress and apoptosis (uPS50-500)
			LO2			5, 10, 25, 50, 100, 250 and 500 µg/mL		↓: Cell viability (uPS100, 500 µg/mL; uPS50, >100 µg/mL; uPS20, >50 µg/mL)
**Li, Y. et al.**	2023a	China	LO2	uPS	**20**, **60**, 100 and 500	1, 5, **25**, 75, and 125 µg/mL	24h	↓: Cell viability[+] (uPS20), cytoplasmic membrane integrity[+] (uPS20/60)<br>↑: Oxidative stress* (uPS20/60), inflammation[#] and apoptosis[#]

Authors	Year	Country	Cellular line	Polymer type	NP size (nm)	NP concentration	Exposure duration	Main findings
								(uPS20)<br>=: Cell viability (uPS100/500), inflammation[#] and apoptosis[#] (uPS60)
**Li, Y. et al.**	2023b	China	HepG2	uPS	20	6.25, 12.5, 25 and **50** μg/mL	24h	↓: Cell viability (>6.25 μg/mL), mitochondrial biogenesis (>12.5 μg/mL)<br>↑: Oxidative stress, apoptosis, and mitochondrial fission (>12.5 μg/mL)
**Paul, M.B. et al.**	2023	Germany	HepaRG	PMMA	25	1×10^9 μm^2 particle surface/mL	24h	No significant negative effects
				PLA	250			↑: Inflammation[#]<br>=: Oxidative stress* and barrier integrity
**Roursgaard, M. et al.**	2022	Denmark	HepG2	PET	< 600 (x = 252)	1, 2, 4, 8, **16**, **32**, **63** ng/mL	24h	↓: Cell viability[b]<br>↑: DNA damage[b] (>16 ng/mL)<br>=: Oxidative stress, cytoplasmic membrane integrity and cell cycle distribution
				PP	< 700 (x = 158)	3, 5, 11, 22, **44**, **88**, **175** ng/mL		No significant changes were found for cell viability, cytoplasmic membrane integrity, DNA damage and cell cycle distribution
**Stock, V. et al.**	2022	Germany	HepaRG	C-PS	20, 40 and 100	$5x10^9$ - $5x10^{11}$ μm^2 particle/mL	24h	↓: Cell viability (C-PS20, >$2.5x10^{11}$ μm^2 particle/mL; C-PS40, $1x10^{12}$ μm^2 particle/mL)<br>↑: Apoptosis (C-PS20, >$2x10^{11}$ μm^2 particle/mL)<br>=: Cell viability (C-PS100) and apoptosis (C-PS40/100)
				A-PS	100	$5x10^9$ - $2.5x10^{11}$ μm^2 particle/mL	24h	↓: Cell viability ($2.5x10^{11}$ μm^2 particle/mL)<br>↑: Apoptosis (>$2x10^{11}$ μm^2 particle/mL)
				uPS				
				S-PS		$1x10^{10}$ - $5x10^{11}$ μm^2 particle/mL		=: Cell viability and apoptosis

Authors	Year	Country	Cellular line	Polymer type	NP size (nm)	NP concentration	Exposure duration	Main findings
**Tolardo, V. et al.**	2022	Italy	HepG2	PC	≈ 47	**1, 10, 20, 40** or 80 μg/mL	24-48h	↓: Cell viability[a] (PC, >10 μg/mL; PET58, >20 μg/mL; PET89, >40 μg/mL)<br>=: Nuclear size and intensity
				PET	≈ 58 and ≈ 89			
**Tolardo, V. et al.**	2023	Italy	2º Gen UHHs	PC	≈ 31.5	**10, 20, 40**, 60, 80 and 100 μg/mL;	24-48h	↓: Cell viability and cytoplasmic membrane integrity (100 μg/mL), and albumin levels (>40 μg/mL)<br>Others: disruption of cytochrome P450 system.
**Zheng, T. et al.**	2019	China	Rat hepatocyte suspensions (C57BL6-J)	uPS	50	**1, 5, 10, 20,** and **30×10^{-6}** mol/L	24h	↑: Oxidative stress (>$10x10^{-6}$ mol/L) and DNA damage (>$5x10^{-6}$ mol/L)

NP polymer type, size, concentration and main findings. In **bold**, values that were carried across in the assessment of different parameters besides cell viability. [+]No statistical analysis. *Based on ROS production. [#]Based on interpretation of mRNA levels and protein levels. [c]Based on decrease of claudin-2 protein levels. MMP = Mitochondrial Membrane Potential. A-PS = Amine-modified Polystyrene, C-PS = Carboxyl-modified Polystyrene, PC = Polycarbonate, PE = Polyethylene, PET = Polyethylene terephthalate, PLA = Polylactic acid, PMMA = Polymethyl methacrylate, PP = Polypropylene, PTFE = Polytetrafluoroethylene, S-PS = Sulfate-modified polystyrene, Sa-PS = Sulfonic acid-modified polystyrene, uPS = unmodified Polystyrene, wPET= weathered Polyethylene terephthalate, wPS = weathered Polystyrene.

3. Summary of urinary cell line studies

Authors	Year	Country	Cellular line	Polymer type	NP size (nm)	NP concentration	Exposure duration	Main findings
**He, S. et al.**	2024	China	HK2	uPS	≈ 158	50, 250, **500**, 750, and 1000 µg/mL	12h	↑: Oxidative stress*<br>=: Cell viability and inflammation#
				wPS	≈ 124			↓: Cell viability (> 750 µg/mL)<br>↑: Cell viability (250 µg/mL), oxidative stress*, inflammation#
**Li, Y. et al.**	2023a	China	293T	uPS	**20**, **60**, 100 and 500	1, 5, **25**, 75, and 125 µg/mL	24h	↓: Cell viability+ (uPS20-100) and cytoplasmic membrane integrity+ (uPS20/60)<br>↑: Oxidative stress* (uPS20/60), inflammation# and apoptosis# (uPS20)
**Xiao, M. et al.**	2023	China	HK2	uPS	50	50, 100, **200**, 500, and 1000 µg/mL	24h	↓: Cell viability+, barrier integrityc<br>↑: Inflammation, oxidative stress and apoptosis#
**Zhu, Z. et al.**	2023	China	HK2	uPS	**20** and 50	**50** and **100** µg/mL	12h	↓: Cell viability (uPS20/50, 100 µg/mL), cytoplasmic membrane integrity (uPS20/50, >50 µg/mL), MMP (100 µg/mL)<br>↑: Oxidative stress* (100 µg/mL), early apoptosis (>50 µg/mL) and late apoptosis (100 µg/mL)

NP polymer type, size, concentration and main findings. In **bold**, values that were carried across in the assessment of different parameters besides cell viability. +No statistical analysis. *Based on ROS production. #Based on interpretation of mRNA levels and protein levels. cBased on decrease of claudin-2 protein levels. MMP = Mitochondrial Membrane Potential. uPS = unmodified Polystyrene. wPS = weathered Polystyrene.

4. **Summary of respiratory cell line studies**

Author	Year	Country	Cellular line	Polymer type	NP size (nm)	NP concentration	Exposure duration	Main findings
**Alzaben, M. et al.**	2023	Denmark	A549	PET	≈ 167	**1.95, 15.6,** and **125** µg/mL	3, 24h	↑: DNA damage[b] and oxidative stress*[b]<br>=: Cell viability and cytoplasmic membrane integrity (≤125 µg/mL)
**Chen, Y.C. et al.**	2023	Hong Kong	BEAS-2B	uPS	≈ 153	**1**, 10, 100 and **1000** ng/cm[2]	24h	↓: Cell viability (>10 ng/cm[2])<br>↑: Oxidative stress* (>1 ng/cm[2]), inflammation[#] (1 ng/cm[2]), apoptosis and autophagy (1000 ng/cm[2])
**Halimu, G. et al.**	2022	China	A549	uPS	50	**40, 80** and **160** µg/mL	24h	↓: Cell viability (uPS50 >40 µg/mL, and uPS20 >20 µg/mL) and MMP (uPS50 160 µg/mL, and uPS20 >40 µg/mL)<br>↑: Oxidative stress* (uPS20/50) and mitochondrial impairment (uPS20/50)
					20	**10, 20** and **40** µg/mL		
				A-PS	20			↓: Cell viability (>20 µg/mL), MMP (>20 µg/mL)<br>↑: Oxidative stress* and mitochondrial impairment
**Huang, J. et al.**	2022	China	HNEpCs	uPS	20, 50, 100, 200 and 500	10, 50, 125, **500**, and 1250 µg/mL	48h	↓: Cell viability: (uPS20/100/500, >125 µg/mL; uPS50, >10 µg/mL; uPS200, 1250 µg/mL)<br>↑: Apoptosis[+] (uPS20-500, 500 µg/mL)
				C-PS	50, 100 and 500			↓: Cell viability (C-PS50/500, >500 µg/mL; C-PS100, >125 µg/mL)
				A-PS				↓: Cell viability (A-PS50/500, >10 µg/mL; A-PS100, >125 µg/mL)<br>↑: Necrosis[+] (A-PS50, 500 µg/mL)
**Lim, S.L. et al.**	2019	Singapore	BEAS-2B	uPS	50	1, 5, **10**, 25, **50**, 75, 100 µg/mL	24h	↓: Cell viability (>10 µg/mL), ATP levels<br>↑: Oxidative stress*, ER stress[#] (50 µg/mL) and autophagy[a]<br>Others: PS NPs interfered with the energy metabolism[&]
**Liu, Y. et al.**	2022	China	A549	uPS	20	5, **10**, 20, 40 and 80 µg/mL	24h	↓ Cell viability (uPS20, >5 µg/mL)

Author	Year	Country	Cellular line	Polymer type	NP size (nm)	NP concentration	Exposure duration	Main findings
**Shi, Q. et al.**	2021	China	A549	uPS	100	10, **20**, 100, **200**, 500 or 1000 µg/mL	24h	↓: Cell viability (>200 µg/mL)<br>↑: Oxidative stress* and inflammation[#] (200 µg/mL)
**Shi, X. et al.**	2022	China	A549	uPS	80	2.5, 5, 10, 25, **50, 100, 200** and **400** µg/mL	6, 9, 24h	↓: Cell viability (>50 µg/mL)<br>↑: DNA damage and oxidative stress* (>100 µg/mL)
				C-PS				↓: Cell viability (>2.5 µg/mL)<br>↑: DNA damage (>50 µg/mL) and oxidative stress* (>100 µg/mL)
				A-PS				
**Soto-Bielicka, P. et al.**	2023	Spain	RTgill-W1	C-PS	40	0.1–200 µg/mL (**10 µg/mL**)	24h	↓: Cell viability (>25 µg/mL)<br>=: Cytoplasmic membrane integrity, MMP, DNA damage and oxidative stress (10 µg/mL)
**Wu, Q. et al.**	2024	China	BEAS-2B	uPS	20	0.05, 0.15, **0.2** mg/mL	24h	↓: Cell viability (>0.15 mg/mL)<br>↑: Oxidative stress*, inflammation[#] and apoptosis (0.2 mg/mL)
**Wu, Y. et al.**	2023a	China	BEAS-2B	uPS	100 and 200	50, **100, 200** and **400** µg/mL	24h	↓: Cell viability (uPS100/200, >100 µg/mL)<br>↑: Oxidative stress* (uPS100/200, >100 µg/mL)<br>Others: PS NP exposure might induce ferroptosis in BEAS-2B cells through the HIF-1α/HO-1 signaling pathway
**Wu, Y. et al.**	2023b	China	MLE-12	A-PS	100	**12.5** µg/mL	12h	↑: Inflammation[#] and apoptosis[#]
			MH-S					↑: Inflammation[#] and oxidative stress[#]
**Xu, M. et al.**	2019	China	A549	uPS	25	2.5, 5, 10, 15, 20, **25** and 30 µg/mL	24h	↓: Cell viability (uPS25, >25 µg/mL and uPS70, >160 µg/mL)<br>↑: Apoptosis (uPS25/70) and inflammation[#] (uPS25/70)<br>Others: Both NPs halted the cell cycle time-dependently at the S phase
					70	10, 30, 60, 100, **160**, 220 and 300 µg/mL		
	2021	China	HPAEpiC	uPS	40		24h	

Author	Year	Country	Cellular line	Polymer type	NP size (nm)	NP concentration	Exposure duration	Main findings
**Yang, S. et al.**			BEAS-2B			8, 16, **24**, 32, **48**, 64, 80, **96**, 112 and 128 µg/mL		↓: Cell viability (>48 µg/mL), cytoplasmic membrane integrity (>24 µg/mL) and barrier integrity (>24 µg/mL)<br>↑: Oxidative stress, inflammation# and apoptosis
**Zhang, H. et al.**	2022	China	A549	PET	≈ 122-221	0.098, 0.98, **4.92**, 9.84, 24.6, **49.2**, 98.4 and 196.76 µg/mL	24h	↓: Cell viability (>98.4 µg/mL); apoptosis (early+late and early)<br>↑: Oxidative stress (>49.2 µg/mL); late apoptosis (196.76 µg/mL)<br>=: MMP

NP polymer type, size, concentration and main findings. In **bold**, values that were carried across in the assessment of different parameters besides cell viability. *Based on ROS production. #Based on interpretation of mRNA levels and protein levels. +No statistical analysis. [b]Based on the slope instead of a single concentration. [a]Based on the increase of LC3-II. &Changes in metabolite profiles, primarily glucose, lactate and alanine. A-PS = Amine-modified Polystyrene. ATP = Adenosine Triphosphate. C-PS = Carboxyl-modified Polystyrene. MMP = Mitochondrial Membrane Potential. PET = Polyethylene terephthalate. uPS = unmodified Polystyrene.

5. **Summary of digestive system cell line studies**

Authors	Year	Country	Cellular line	Polymer type	NP size (nm)	NP concentration	Exposure duration	Main findings
**Banaei, G. et al.**	2023	Spain	Co-culture (Caco-2/HT29)	PLA	≈ 280	**50** and **100** μg/mL	48-72h	↓: Barrier integrity (3h, 50 μg/mL)<br>=: Cell viability, oxidative stress*, barrier permeability and barrier integrity (>3h)
**Banerjee, A. et al.**	2021	USA	SNU-1	uPS<br>C-PS<br>A-PS	50, 100, 200 and 500	0.1-**100** μg/mL	24h	↓: Cell viability (uPS50, ≥75 μg/mL; A-PS50, >7.5 μg/mL; A-PS100, >50 μg/mL; A-PS500, >10 μg/mL)<br>=: Cell viability (C-PS50-500, A-PS200 and uPS100-500)<br>Others: No other comparison to control was made; comparison between particles reveals higher apoptotic/necrotic effects in A-PS particles and smaller particles (50 nm)
**Brandts, I et al.**	2023	Spain	RTgutGC	uPS	44	**25** and **50** μg/mL	1, 12 and 16h	No significant differences for cell viability, respiratory capacity and oxidative stress* were observed
**Busch, M. et al.**	2021	Germany	Caco-2	uPS<br>A-PS	50	1, 5, **10** or **50** μg/cm²	24h	↓: Cell viability (A-PS, >1 μg/cm²) and cytoplasmic membrane integrity (A-PS, 50 μg/cm²)<br>↑: DNA damage (A-PS, 50 μg/cm²)<br>=: Cell viability, cytoplasmic membrane integrity and DNA damage (uPS)
			HT29-MTX-E12					↓: Cell viability (A-PS, >10 μg/cm²) and cytoplasmic membrane integrity (A-PS, >10 μg/cm²)<br>↑: DNA damage (A-PS, 50 μg/cm²)<br>=: Cell viability, cytoplasmic membrane integrity and DNA damage (uPS)

			Triculture (Caco-2/HT29/THP-1)					↓: Cytoplasmic membrane integrity (A-PS, 50 µg/cm^2)<br>=: Cytokine release (uPS)
**Chen, W. et al.**	2021	China	Caco-2	uPS	≈ 80	3.125, 6.25, 12.5, 25, 50, **100** and 200 µg/mL	24h	↓: Cell viability (>100 µg/mL) and impaired autophagic flux<br>↑: Oxidative stress*
**Cortés, C. et al.**	2020	Spain	Caco-2	uPS	40-100	**1, 25, 50, 100**, 125, 150, 175, 200 µg/mL	24-48h	↓: MMP (1 µg/mL)<br>↑: MMP and oxidative stress response[#]<br>=: Cell viability, inflammation[#], oxidative stress* and DNA damage<br>Others: At the highest concentration (200 µg/mL) there was a decrease of cell viability to 80% relative to control[+]
**Cui, M. et. al**	2023	China	Co-culture (Caco-2/HT29-MTX)	C-PS	**20** and 200	**1, 10, 100**, 200, 400, 800, **1000**, 1200, 1600 and 2000 µg/mL	24-48h	↓: Cell viability (C-PS20, Caco-2, >1000 µg/mL; HT29-MTX, >400 µg/mL; and co-culture >1600 µg/mL) and barrier integrity (C-PS20, >100 µg/mL)<br>↑: Oxidative stress* (>100 µg/mL) and model's mucus secretion (>10 µg/mL)<br>=: Cell viability (C-PS200, Caco-2, HT29-MTX and co-culture)<br>Others: HO1/p38/IL-10 axis was involved in the MUC2 induction and the increased mRNA levels of HO-1 and IL10 were due to Caco-2 cells
**DeLoid, G.M. et al.**	2021	USA	Triculture small intestinal epithelial model (Caco-2, HT29-	uPS	25	**0.4** and **1** mg/mL	4 and 24h	=: Cell viability, barrier integrity, membrane permeability, cytoplasmic membrane integrity and oxidative stress*
				C-PS	25 and 100			↓: Cell viability (C-PS25, 1 mg/mL; C-PS100, >0.4 mg/mL)<br>↑: Membrane permeability (PS100C, 0.4 mg/mL)<br>=: Barrier integrity, cytoplasmic membrane integrity and oxidative stress* (C-PS25/100)

			MTX and M-cells)					
**Ding, R. et al.**	2024	China	GES-1	uPS	80, 200 and 500	12.5, 25, **50**, 100 and 200 μg/mL	24h	↓: Cell viability (uPS80, >50 μg/mL; uPS200/500, > 100 μg/mL)<br>↑: Oxidative stress* (uPS80-500) and DNA damage[#+] (uPS80-500)<br>Others: activation of the β-catenin/YAP cascade
**Ding, Y. et. al**	2021	China	GES-1	uPS	60	**50** μg/mL	2, 4, 6, 12, 24 and 48h	↓: Cell proliferation (24/48h); MMP (12/24h)<br>↑: Apoptosis ratio (12/24h), autophagosomes and autolysosomes formation (12-48h)<br>=: Apoptosis (48h)
**Domenech, J. et al.**	2020	Spain	Co-culture (Caco-2/HT29)	uPS	40-100	**1, 25, 50, and 100** μg/mL	24h	=: Cell viability, cytoplasmic membrane integrity, DNA damage and oxidative stress*[#]
**Domenech, J. et al.**	2021 a	Spain	Caco-2	uPS	50	0.0006, 0.26, 1.3 and 6.5 μg/cm^2	8 weeks	↑: Oxidative stress response[d] (8 weeks) and DNA damage (8 weeks, 0.26 μg/cm^2)<br>=: Cell viability, oxidative stress* (24h and 8 weeks) and DNA damage (24h)
						**0.26, 6.5**, 13, 26, and 39 μg/cm^2	24h	
**Domenech, J. et al.**	2021 b	Spain	Caco-2	uPS	40-100	**10**, 25, 50, **100**, 125, 150, 175, and 200 μg/mL	24h	=: Cell viability, oxidative stress* and DNA damage
**Guanglin, L. et al.**	2024	China	HET-1A<br>HEEC	uPS	100	10, **30**, and 50 μg/mL	24h-72h	↓: Cell viability (>10 μg/mL)<br>↑: Inflammation[#], apoptosis (>10 μg/mL), oxidative stress* (30 μg/mL)<br>Others: Cell inflammation and death may be caused by Fe^{2+} accumulation
**He, Y.J. et al.**	2022	China	NCM460	uPS	100	**50** μg/mL	24h	↓: barrier integrity[#]<br>↑: Oxidative stress* and inflammation[#]

**Hesler, M. et al.**	2019	Germany	Co-culture (Caco-2/HT29-MTX-E12)	C-PS	50	0.01, 0.1, 1, 5, **10**, 25, 50 and **100** µg/mL	24-48h	↓: Cell viability (100 µg/mL)<br>=: Barrier integrity
**Hou, Z.K. et al.**	2022	China	Human intestinal organoid culture from HiPSCs	uPS	50	**10**, 50, 100, 150, and 200 µg/mL	Cell viability: 24-48h; Others: 14 days	↓: Cell viability (48h, >150 µg/mL)<br>↑: Apoptosis, oxidative stress* and inflammation#<br>=: Cell viability (24h, ≤200 µg/mL)
**Jin, M.H. et al.**	2023	China	Caco-2	uPS	80	50, 100, 200, 300, 400, **500**, 600 and 700 µg/mL	24h	↓: Cell viability (>400 µg/mL), MMP, barrier integrity and lysosomal function<br>↑: Oxidative stress*, apoptosis and autophagy#
**Kaur, J. et al.**	2023	Sweeden	HT-29	C-PS	200	0.1–**100** µg/mL	24h	↓: Barrier integrity (C-PS, 100 µg/mL, with or without FBS)<br>=: Cell viability
				A-PS				=: Barrier integrity and cell viability
**Li, C. et al.**	2023	China	Caco-2	uPS	100	**100** and **200** µg/mL	48h	=: Cell viability, inflammation and oxidative stress#
**Liu, Y. et al.**	2022	China	Caco-2	uPS	20 and 100	5, **10**, 20, 40 and 80 µg/mL	24h	↓: Cell viability (uPS20, >5 µg/mL and uPS100, >10 µg/mL) and MMP (uPS20/100, 10 µg/mL)<br>↑: Oxidative stress* (uPS20/100, 10 µg/mL)<br>=: Cytoplasmic membrane integrity (uPS20/100, 10 µg/mL)
**Magrì, D. et al.**	2018	Italy	Caco-2	PET	≈ 26,7	**1, 5, 15,** and **30** µg/mL	24, 72, and 96 h	=: Cell viability, cytoplasmic membrane integrity, apoptosis/necrosis, oxidative stress* and inflammation#
**Magrì, D. et al.**	2021	Italy	Caco-2	PET	10-80 (x = 30)	**30** µg/mL	24-96h	↓: Alanine production and lactate/glucose ratio<br>↑: Essential and non-essential aminoacids' consumption (phenylalanine, threonine, lysine, valine, methyonine, glycine, tyrosine and glutamine) and lactate/alanine ratio

								=: Cell viability, oxidative stress* and acetate consumption
**Paul, M.B. et al.**	2023	Germany	Caco-2	PMMA	25	**1×10^9** µm^2 particle surface/mL	24h	No consistent significant effects were observed but there was an increase in NFkB and CAT with a decrease in GSTP1 indicating possible response to oxidative stress/inflammation
				PLA	250			↑: Inflammation[#]<br>=: Oxidative stress* and barrier integrity
**Peng, M. et al.**	2024	Belgium	Caco-2	wPET	<800 (x = 144)	10^2 to 10^7 particles/mL	48h	↑: ECAR and glycolytic ATP
				PET	<800 (x = 197)			↑: OCR, ECAR and mitochondrial ATP
				wPS	100+750			No significant differences were found in OCR, ECAR, mitochondrial or glycolytic ATP
				uPS				↑: ECAR
**Roursgaard, M. et al.**	2022	Denmark	Caco-2	PET	<600 (x = 252)	1, 2, 4, 8, **16, 32** and **63** ng/mL	24h	↓: Cell viability[b] and cytoplasmic membrane integrity[b]<br>↑: DNA damage[b] (>16 ng/mL)<br>=: Oxidative stress* (3h) and cell cycle distribution
				PP	<700 (x = 158)	3, 5, 11, 22, **44, 88** and **175** ng/mL		=: Cell viability[#], cytoplasmic membrane integrity[#], DNA damage, oxidative stress* (3h) and cell cycle distribution
**Sun, R. et al.**	2024	China	GES-1	uPS	50	10, **20, 40** and **80** µg/mL	24-48h	↓: Cell viability (>20 µg/mL) and MMP (80 µg/mL)<br>↑: Apoptosis (early, late and total), mitochondrial dysfunction[#] and oxidative stress* (>40 µg/mL)
					250			↓: Cell viability (>40 µg/mL)<br>↑: Early apoptosis (80 µg/mL) and oxidative stress* (>40 µg/mL)<br>=: MMP, late and total apoptosis
**Tolardo, V. et al.**	2022	Italy	Caco-2	PC	≈ 47	**1, 10, 20, 40** or 80 µg/mL	24-48h	↓: Cell viability (>10 µg/mL) and mitochondrial activity (80 µg/mL)<br>=: Nuclear size and intensity<br>Others: EC50 at 48h was 44.62 µg/mL and the dispersant also caused a significant decrease in cell viability

				PET1	≈ 58			↓: Cell viability (80 μg/mL)<br>↑: Cell viability (10 and 20 μg/mL)<br>=: Nuclear size and intensity<br>Others: EC50 at 48 was 40.06 μg/mL and the dispersant also caused a significant decrease in cell viability
				PET2	≈ 89			↓: Cell viability (>20 μg/mL)<br>=: Nuclear size and intensity<br>Others: EC50 at 48h was 82.12 μg/mL
**Xu, D. et al.**	2021	China	Caco-2	uPS<br>C-PS<br>A-PS	100	**30**, 60, 120, 240, 480 μg/mL	24, 48 or 96h	↓: Cell proliferation (24h, uPS100, 480 μg/mL; 24h, A-PS100, >60 μg/mL; 96h, all polymers, >30 μg/mL) and barrier integrity<br>↑: Cell arrest in G0/G1 phase, apoptosis and n° of lysosomes per cell (48h, 30 μg/mL)<br>=: Cytoplasmic membrane integrity (30 μg/mL)
**Xu, X. et al.**	2023	China	RKO	uPS	100	1, **10**, 50, 100 μg/mL	24-48h	↓: Cell viability (48h, >50 μg/mL)<br>↑: Apoptosis (100 μg/mL) and impaired autophagic flux (due to increase of LC3II and p62)
			HT-29					↓: Cell viability (48h, >50 μg/mL)<br>↑: Apoptosis (100 μg/mL)
			HCT-116					↓: Cell viability (48h, >50 μg/mL)<br>↑: Apoptosis (>50 μg/mL)
			HIEC-6					↓: Cell viability (24h/48h, >10 μg/mL)<br>↑: Apoptosis (>10 μg/mL) and autophagy (LC3II increase)
**Xuan, L. et al.**	2024	China	Mouse intestinal organoids	uPS<br>PTFE<br>PMMA	100<br>≈ 230<br>≈ 150	**50** μg/mL	3 days	↓: MMP (all polymers, both models)<br>↑: Oxidative stress, apoptosis and necrosis (all polymers, both models) and inflammation (all polymers on mouse intestinal organoids)<br>Others: Inhibition of the AKT-mTOR signaling pathway; overall uPS had the biggest effect and PMMA the lowest
			HCT116			**100** μg/mL	Not disclosed	
**Yan, L. et al.**	2023	China	Caco-2	uPS	20	0.1, **10**, 50, 100, 500 μg/mL	24h	↓: Cell viability (>10 μg/mL)<br>=: Oxidative stress*, cytoplasmic membrane integrity, cell membrane fluidity and MMP

								Others: Complex cellular response to NP exposure that might lead to apoptosis[#]
**Zhang, Y. et al.**	2024	China	IEC-6	uPS	≈ 80	**25, 50**, 75, and 100 μg/mL	24, 48h	↓: Cell viability (48h, >25 μg/mL), MMP (>25 μg/mL) and cytoplasmic membrane integrity (>50 μg/mL)<br>↑: Oxidative stress*, mitochondrial damage and autophagy[#]

NP polymer type, size, concentration and main findings. In bold, values that were carried across in the assessment of different parameters besides cell viability. *Based on ROS production. [#]Based on interpretation of mRNA levels and protein levels. [+]No statistical analysis. [d]Based on HO-1 protein levels and SOD activity. [b]Based on the slope instead of a single concentration. [d]Based on gene expressions of HO-1 and SOD2. A-PS = Amine-modified Polystyrene. CAT = Catalase. C-PS = Carboxyl-modified Polystyrene. EC50 = Half Maximal Effective Concentration. ECAR = Extracellular Acidification Rate. FBS = fetal bovine serum. GST = Glutathione S-Transferase. MMP = Mitochondrial Membrane Potential. OCR = Oxygen Consumption Rate. PC = Polycarbonate. PET = Polyethylene terephthalate. PLA = Polylactic Acid. PMMA = Polymethyl methacrylate. PP = Polypropylene. PTFE = Polytetrafluoroethylene. uPS = unmodified Polystyrene. wPET= weathered Polyethylene terephthalate. wPS = weathered Polystyrene.

6. Summary of immune system cell line studies

Authors	Year	Country	Cellular line	Polymer type	NP size (nm)	NP concentration	Exposure duration	Main findings
**Babonaitė, M. et al.**	2023	Lithuania	PBMCs	uPS	50-100	15, 20, **25**, 30, 40, **50**, 60, **75**, 85 and **100** µg/mL	3, 24h	**↓:** cell viability (60 and 85 µg/mL)<br>↑: DNA damage (24h, >15 µg/mL)<br>**=:** cell viability (all other concentrations)
**Ballesteros, S. et al.**	2020	Spain	WBCs	uPS	40-100	1, 10, 25, **50**, and **100** µg/mL	24, 48 and 72h	**↑:** DNA damage (PMNs and monocytes, 100 µg/mL) and inflammation[#]<br>**=:** cell viability; DNA damage (lymphocytes)
**Brandts, I et al.**	2023	Spain	RT-HKM	uPS	44	**25** and **50** µg/mL	1, 12 and 16h	=: Cell viability and oxidative stress*<br>Others: induction of a specific polarization state (different than M1 and M2-like phenotypes)
**Busch, M. et al.**	2021	Germany	THP-1	uPS	50	1, 5, **10** or **50** µg/cm^2	24h	=: Cell viability and cytoplasmic membrane integrity
				A-PS				↓: Cell viability (>5 µg/cm^2) and cytoplasmic membrane integrity (>5 µg/cm^2)<br>↑: expression of IL-1β (5-10 µg/cm^2)<br>=: expression of IL-1β (50 µg/cm^2) and cytokine release (on triple culture with Caco-2/HT29-MTX-E12)
**Chen, J. et al.**	2023	China	RAW264.7	uPS	100	0, 1, 2, 5, **10**, **20**, **50** and **100** µg/mL	6-24h	↓: MMP<br>↑: Oxidative stress* (>10 µg/mL)<br>=: Cell viability, cytoplasmic membrane integrity and apoptosis
				C-PS				↓: Cell viability (>20 µg/mL) and MMP<br>↑: Oxidative stress* (>10 µg/mL) and apoptosis (>20 µg/mL)<br>=: Cytoplasmic membrane integrity

Authors	Year	Country	Cellular line	Polymer type	NP size (nm)	NP concentration	Exposure duration	Main findings
				A-PS				↓: Cell viability (>1 µg/mL), cytoplasmic membrane integrity (>2 µg/mL) and MMP<br>↑: Oxidative stress* (>10 µg/mL) and apoptosis (>10 µg/mL)
**Djapovic, M. et al.**	2023	Serbia	PBMCs	PET	≈ 300	0.001, 0.01, 0.1, **1, 10** and **100** µg/mL	4, 24h	**=:** cell viability, apoptosis and oxidative stress***.**
**Florance, I. et al.**	2021	India	RAW264.7	uPS	200	1, 5, 10, 25, **50**, **100** and **200** µg/mL	24-96h	↑: Oxidative stress* (200 µg/mL)<br>= Cell viability (96h, ≤ 200 µg/mL)
				S-PS				↑: Foam cell formation (24h, 200 µg/mL)<br>= Cell viability (96h, ≤ 200 µg/mL) and oxidative stress*
**Florance, I. et al.**	2022	India	RAW 264.7	S-PS	200	5, 10, 25, **50, 100**, 250 and 500 µg/mL	24h	↓: Cell viability (>250 µg/mL) and MMP<br>↑: Lipidic content and mtROS (100 µg/mL)
			THP-1					↓: MMP<br>↑: Lipidic content and mtROS (100 µg/mL)<br>=: Cell viability
**Giannandrea, D. et al.**	2024	Italy	RAW264.7	uPS	50	1, 10, **50, 100** and 200 µg/ml	4-96h<br>Osteoclastic differentiation assay: 7 days	↓: Cell viability (>10 µg/mL)<br>↑: apoptosis[#] (>100 µg/mL) and oxidative stress*<br>Others: Exposure increased differentiation of RAW264.7 toward osteoclast cells
**Guo, X. et al.**	2023	China	Human HSPCs	uPS	80	0.05, **0.1**, 0.2, 0.4, and 0.6 mg/mL	12-48h	↓: Cell viability (>0.2 mg/mL), cytoplasmic membrane integrity (>0.1 mg/mL) and colony growth of CFU-GM, CFU-E and BFU-E<br>↑: Oxidative stress*

Authors	Year	Country	Cellular line	Polymer type	NP size (nm)	NP concentration	Exposure duration	Main findings
								=: Colony growth of GEMM<br>Others: 10 metabolites altered out of 176 tested
**Ilić, K. et al.**	2022	Croatia	Jurkat cells	uPS	20	1, **10**, 50, **100**, 250, 500 mg/L	24h	**↓:** cell viability; MMP (all except 10 mg/L without FBS)<br>**↑:** apoptosis (with FBS, 500 mg/L; withouth FBS, >50 mg/L) and oxidative stress* (withouth FBS, >10 mg/L)<br>**=:** Oxidative stress* (with FBS)
**Li, C. et al.**	2023	China	THP-1	uPS	100	**100** and **200** µg/mL	48h	=: Cell viability and inflammation[e]
**Li, S. et al.**	2023	China	RAW264.7	uPS	80	**400** µg/mL;	24h	↓: CD206<br>↑: Oxidative stress* and inflammation[#]
**Li, Y.Q. et al.**	2022	China	Splenocytes from female wt BALB/c mice	uPS	20 and 50	PS20: 5, 10, 20, **40**, 80 and 160 µg/mL<br><br>PS50: 25, 50, 100, **200**, 400 and 800 µg/mL	6-24h	↓: Cell viability (uPS20 >40 µg/mL and uPS50 >200 µg/mL) and MMP (uPS20/50)<br>↑: Apoptosis (uPS20 >10 µg/mL and uPS50 >200 µg/mL) and oxidative stress* (uPS20/50)
				Sa-PS	20			↓: Cell viability (>40 µg/mL) and MMP<br>↑: Apoptosis (>20 µg/mL) and oxidative stress*
				A-PS				↓: Cell viability (>10 µg/mL) and MMP<br>↑: Apoptosis (>10 µg/mL)<br>=: Oxidative stress*
**Murano, C. et al.**	2021	Italy	Primary cultures of coelomocytes	C-PS	60	**5** and **25** µg/mL	4h	↓: Lysosomal membrane stability and phagocytic capacity (25 µg/mL)<br>=: Cell viability

Authors	Year	Country	Cellular line	Polymer type	NP size (nm)	NP concentration	Exposure duration	Main findings
				A-PS	50	**25** µg/mL		↓: Cell viability, lysosomal membrane stability, phagocytic capacity and phagocytic index (25 µg/mL)
**Nikolic, S. et al.**	2022	Serbia	Splenocytes	C-PS	40+200	**0.01** and **0.1** mg/mL	24-72h	**↓:** cell viability (72h, both concentrations)<br>**↑:** DNA damage and apoptosis
**Rubio, L. et al.**	2020	Spain	THP-1	uPS	50-100	0-200 µg/mL (**5, 10, 25** and **50** µg/mL)	3, 24, 48h	=: Cell viability, oxidative stress* and DNA damage
			TK-6<br>Raji-B					↓: Cell viability (Both cell lines >200 µg/mL)<br>↑: Oxidative stress* (TK6, 24h, >5 µg/mL) and DNA damage (both cell lines)<br>=: Oxidative stress* (Raji-B, 24h)
**Tan, Y. et al.**	2020	China	RAW264.7	uPS	300	50, 100, **150** and 200 µg/mL	24h	↓: Cell viability (>50 µg/mL) and cytoplasmic membrane integrity<br>↑: Lysosomal membrane permeability<br>Others: NP exposure disrupted the autophagic flow despite the upregulation of LC3B-II
**Tavakolpournegari, A. et al.**	2023	Spain	TK6<br>THP-1<br>Raji-B	uPS	50, 200 and 500	**50, 100**, 150, 200 µg/mL	3, 24 and 48 h	↓: MMP (THP-1, uPS200/500, >50 µg/mL; THP-1, uPS50, 100 µg/mL; Raji-B, uPS200/500, 100 µg/mL; Raji-B, uPS50, 50 µg/mL)<br>=: Cell viability (all cell lines), oxidative stress* after 48h (all cell lines) and MMP (TK6)
**Vela, L. et al.**	2023	Spain	TK6	uPS	40-100	50, 100, 150 and 200 µg/mL	24-48h	↓: Cell viability (digested and normal uPS)<br>↑: Oxidative stress*<br>=: DNA damage

Authors	Year	Country	Cellular line	Polymer type	NP size (nm)	NP concentration	Exposure duration	Main findings
			THP-1			(**5, 10, 25** and **50** µg/mL)		No significant changes to cell viability, oxidative stress* or DNA damage
			Raji-B					↓: Cell viability (uPS)<br>↑: Oxidative stress*<br>=: DNA damage
**Wang, X. et al.**	2023	China	RAW264.7	uPS	80	**0.01**, **0.1**, 0.5, **1**, 5 and **10** µg/mL	24h	**↓:** cell viability (>0.1 µg/mL)<br>**↑:** necrosis (0.1 µg/mL), apoptosis (5 µg/mL), oxidative stress* (>1 µg/mL) and inflammation[#]
**Yang, Q. et. al**	2023	China	RAW264.7	PE	≈ 531	5, 10, **20** and 50 µg/mL	12-24h	↑: Lysosomal damage and inflammation[#]<br>=: Cell viability (≤50 µg/mL)
**Vela, L. et al.**	2023	Spain	TK6	uPS	40-100	50, 100, 150 and 200 µg/mL<br>(**5, 10, 25** and **50** µg/mL)	24-48h	↓: Cell viability (digested and normal uPS)<br>↑: Oxidative stress*<br>=: DNA damage

NP polymer type, size, concentration and main findings. *Based on ROS production. In **bold**, values that were carried across in the assessment of different parameters besides cell viability. [#]Based on interpretation of mRNA levels and protein levels. [e]Based on cytokine secretion and NF-kB p65 protein levels. A-PS = Amine-modified Polystyrene. C-PS = Carboxyl-modified Polystyrene. FBS = Fetal Bovine Serum. MMP = Mitochondrial Membrane Potential. PBMCs = Peripheral Blood Mononuclear Cells. PE = Polyethylene. PET = Polyethylene terephthalate. Sa-PS = Sulfonic acid-modified Polystyrene. S-PS = Sulfate-modified Polystyrene. uPS = unmodified Polystyrene.

7. Summary of reproductive system cell line studies

Authors	Year	Country	Cellular line	Polymer type	NP size (nm)	NP concentration	Exposure duration	Main findings
**Basini, G et al.**	2021	Italy	Swine granulosa cells	uPS	100	**5, 25,** and **75** μg/mL	48h	↓: P4 production (>25 μg/mL)<br>↑: cell proliferation, E2 production and oxidative stress<br>=: cell viability
**Chen, G. et al.**	2023	China	HEY	uPS	100	0, **1**, 2.5, **5**, **7.5**, **10**, 12.5, 15, **20**, 30, 40 mg/L	24-48h and 16 days	↓: Cell viability and percentage of wound healing (20 mg/L)<br>Others: EC50 was 31,5 mg/L at 48h and 30 mg/L at 16 days. 275 DEGs were found (110 up-regulated and 165 down-regulated)
**Contino, M. et al.**	2023	Italy	Spermatozoa	A-PS	50 and 100	**0.1, 0.5** and **1** μg/mL	30min.	↓: Motility (A-PS50, >0.5 μg/mL); cytoplasmic membrane integrity (A-PS50, 1 μg/mL); mitochondria functionality (A-PS50, >0.1 μg/mL)<br>↑: Acrosomal damage (A-PS50/100, 1μg/mL); DNA damage (A-PS50); oxidative stress* (A-PS50, >0.5 μg/mL and A-PS100, >0.1 μg/mL)<br>=: motility, cytoplasmic membrane integrity, DNA damage and mitochondria functionality (A-PS100)
**González-Fernández, C. et al.**	2018	France	Oysters' spermatozoa	A-PS<br>C-PS	100	**0.1, 1, 10** and **100** mg/L	5h	↓: Single spermatozoa and spermatozoa aggregates (C-PS, 100 mg/L)<br>↑: cellular relative complexity (C-PS and A-PS, >10 mg/L)<br>=: spermatozoa motility
			Oysters' oocytes					↑: Oocyte mortality (C-PS and A-PS, 100 mg/L) and oxidative stress* (C-PS, 100 mg/L)<br>=: Oocyte cell number, relative size and complexity (C-PS and A-PS)

**Hu, R. et al.**	2022	China	TM4	uPS	20	0, 12.5, 25, **50**, 100 and 200 μg/mL	0, 3, 6, 12, **24h**	↓: cell viability (24h, >100 μg/mL), cytoplasmic membrane integrity (>25 μg/mL), barrier integrity<br>↑: ER stress and UPR
**Li, S. et al.**	2022	China	GC2	uPS	80	**400** μg/mL	24h	↓: MMP<br>↑: Oxidative stress, apoptosis and autophagy[#]
**Li, S. et al.**	2023	China	GC2	uPS	80	**400** μg/mL	24h	↓: MMP (co-culture with RAW264.7)<br>↑: Apoptosis (co-culture), oxidative stress* (monoculture and co-culture) and inflammation[#]
**Ma, T. et al.**	2023	China	TM4	uPS	100	**30** and **300** μg/mL	24h	↓: Cell viability (300 μg/mL) and autophagy[#]
**Ruan, Y. et al.**	2023	China	HeLa	uPS	10, 15, 25, 40 and 50	0, 1, 10, 20, 40, 80, **100**, 200 and 500 mg/L	1, 4h	↓: cell viability (uPS10, >40 mg/L; uPS15, >80 mg/L)<br>↑: Oxidative stress* (uPS10, >20 mg/L; uPS15, >10 mg/L)
**Sui, A. et al.**	2023	China	TM3	uPS	20	0, **40**, 80, 120 and 160 μg/mL	0, 6, **12**, 24h	↓: Cell viability (>120 μg/mL)<br>↑: Oxidative stress* and translation of HIF-1α mRNA via ERK1/2 MAPK/mTOR/4E-BP1 and AKT/mTOR/4E-BP1 pathways.
**Sun, Z. et al.**	2023	China	TM3	uPS	20	**50, 100** and **150** μg/mL	24h	↓: Cell viability, MMP and steroid hormone biosynthesis/metabolism[#] (>50 μg/mL), testosterone levels and cytoplasmic membrane integrity (>100 μg/mL)<br>↑: Oxidative stress* (>50 μg/mL) and apoptosis (>100 μg/mL)
**Xiao, M. et al.**	2023	China	NTERA-2	uPS	50	50, 100, **200**, 500, and 1000 μg/mL	24h	↓: Cell viability (36/48h, >200 μg/mL)<br>↑: Apoptosis[#], oncogenic potential through activation of MAPK and PI3K-AKT signaling pathways
**Zeng, L. et. al**	2023	China	KGN	uPS	20	50 μg/mL, **100 μg/mL** and 200 μg/mL	48h	↓: cell viability (>100 μg/mL)<br>↑: Oxidative stress* and apoptosis (48h, 100 μg/mL), protein levels in the Hippo signaling pathway.

NP polymer type, size, concentration and main findings. In **bold**, values that were carried across in the assessment of different parameters besides cell viability. *Based on ROS production. [#]Based on interpretation of mRNA levels and protein levels. A-PS = Amine-modified Polystyrene. C-PS = Carboxyl-modified Polystyrene. DEG = Differentially Expressed Genes. E2 = Estradiol. ER = Endoplasmic reticulum. MMP = Mitochondrial Membrane Potential. P4 = Progesterone. UPR = Unfolded Protein Response. uPS = unmodified Polystyrene.

8. **Summary of gestational tissue cell line studies**

Authors	Year	Country	Cellular line	Polymer type	NP size (nm)	NP concentration	Exposure duration	Main findings
**Dusza, H.M. et al.**	2022	Netherlands	BeWo b30 (syncytialized and non-syncytialized)	uPS and wPS	50 and 200	0.1, 1, **10**, and 100 µg/mL	24h	↓: cytoplasmic membrane integrity (non-syncytialized cells, uPS50 and wPS50, 100 µg/mL)<br>=: cell viability (syncytialized and non-syncytialized cells, uPS50/200 and wPS50/200) and cytoplasmic membrane integrity (syncytialized cells uPS50/wPS50; both cells uPS200/wPS200)
**Fu, Y. et al.**	2022	China	HUVEC	uPS	50	5, **10**, 15, **20**, and 25 µg/mL	0, 24, 48, and 72 h	↓: MMP^+<br>↑: Oxidative stress* (>10 µg/mL)<br>No significant changes to cell viability or cytoplasmic membrane integrity
				A-PS				↓: Cell viability (>5 µg/mL), cytoplasmic membrane integrity (>10 µg/mL) and MMP^+<br>↑: Oxidative stress* (20 µg/mL)<br>Others: mitochondrial gene expression changed mostly with A-PS NPs and/or 20 µg/mL of any polymer tested
**Hesler, M. et al.**	2019	Germany	BeWo b30	C-PS	50	0.01, 0.1, 1, 5, **10**, 25, 50 and **100** µg/mL	24-48h	↓: cell viability (>5 µg/mL)<br>=: barrier integrity; DNA damage
**Hu, J. et al.**	2022	China	HTR8/SVneo	uPS	100	10, 50, or **100** µg/mL	12, 24h	↓: trophoblast migration and invasion (100 µg/mL)<br>↑: Oxidative stress* (100 µg/mL), apoptosis and inflammation<br>=: cell proliferation<br>Others: 344 DEGs (46 up-regulated and 298 down-regulated)

NP polymer type, size, concentration and main findings. In **bold**, values that were carried across in the assessment of different parameters besides cell viability. *Based on ROS production. +No statistical analysis. A-PS = Amine-modified Polystyrene. C-PS = Carboxyl-modified Polystyrene. DEG = Differentially Expressed Genes. MMP = Mitochondrial Membrane Potential. uPS = unmodified Polystyrene. wPS = weathered Polystyrene.

9. Summary of nervous system cell line studies

Authors	Year	Country	Cellular line	Polymer type	NP size (nm)	NP concentration	Exposure duration	Main findings
**Almeida, M. et al.**	2019	Portugal	DLB-1	uPS	100	**0.001, 0.01, 0.1, 1** and **10** mg/L	24h	↑: GST activity (potential increase in oxidative stress)<br>=: cell viability and CAT activity
**Bai, H. et al.**	2024	China	hCMEC/D3	A-PS	100	3.725, 6.25, 12.5, 25, **40** and **50** µg/mL	12h	↓: Cell viability (>12.5 µg/mL) and barrier integrity[#]
			HT22					↓: Cell viability (>20 µg/mL)<br>↑: apoptosis[#]<br>Others: activation of GAPDH/Ac-Tau signaling
**Ban, M. et al.**	2021	Japan	SH-SY5Y	uPS	50	**2, 10** and **50** µL/mL	24h	↓: cell viability (>10 µL/mL); nº and length of processes<br>↑: cell nucleus staining with DAPI (50 µL/mL)<br>Others: disorderly granular substances were observed in the 10 and 50 µL/mL PS addition groups.
**González-Fernández, C. et al.**	2021	Spain	SaB-1	uPS	50	0.001 to 100 (**1** and **12** µg/mL)	24h	No significant changes to cell viability
				C-PS				
				A-PS				↓: Cell viability<br>↑: Oxidative stress and apoptosis[#]
**Huang, Y. et al.**	2023	China	SH-SY5Y	uPS	50	**0.5, 5, 50** and **500** µg/mL	48h	↓: Cell viability (>50 µg/mL) and MMP (>5 µg/mL)<br>↑: Oxidative stress* (>50 µg/mL), mitochondrial dysfunction[f] and autophagy/mitophagy[#]
				PE	100			No significant changes were observed in any of the effects tested
**Jeong, B. et al.**	2022	South Korea	Primary hippocampal NSCs from E16.5 mice	uPS	50 and 500	5, 10, **25**, 50, 100 µg/mL	7-9 days	↓: Cell proliferation, number of cells, cell diameter and Tuj1+ neuron's length (all polymers)<br>↑: Number of GFAP+ astrocytes (all polymers)<br>Others: Downregulation of genes related to cell division and proliferation
				C-PS				

Authors	Year	Country	Cellular line	Polymer type	NP size (nm)	NP concentration	Exposure duration	Main findings
**Liu, S. et al.**	2022	China	HT22	uPS	100	**5, 25** and **75** µg/mL	24h	↓: cell viability (75 µg/mL from static exposure; >25 µg/mL during dynamic exposure)<br>↑: Oxidative stress* (24h, >5 µg/mL)<br>=: apoptosis, cell cycle phase distribution.
**Martin-Folgar, R. et al.**	2024	Spain	hNS1	uPS	30	**0.5, 2.5,** and **10** µg/mL	4 days	Multifaceted cellular response to NP exposure, involving alterations in the transcriptional level of genes related to oxidative stress responses, DNA repair mechanisms, inflammation, and apoptosis regulation.
**Ruiz-Palacios, M. et al.**	2020	Spain	FuB-1	uPS	100	10^{-7} up to 10 mg/L	24h	↓: Cell viability<br>Others: Compromised oxidative stress response and detoxification capacity[g] and LD50 of 11.24 mg/L
**Shan, S. et al.**	2022	China	hCMEC/D3	uPS	42	25, 50, **100** and 200 µg/mL	72h	↓: Cell viability (200 µg/mL) and barrier integrity<br>↑: Oxidative stress and necrosis<br>=: apoptosis
			BV2			**100** µg/mL	24h	↑: Inflammation[#]
			HT22					↓: Cell viability (100 µg/mL)
**Sun, J. et al.**	2023	China	BV2	uPS	44	**25, 50** and **100** µg/mL	12-24h	↓: Cell viability (>25 µg/mL)<br>↑: Apoptosis, inflammation[#] and oxidative stress*[#]
**Yang, S. et al.**	2023	South Korea	C17.2	uPS	50	100, **200**, or 500 µg/mL	48h	No significant changes in cell viability or oxidative stress were observed
				C-PS				
				A-PS	30			↓: Cell viability (>100 µg/mL)<br>↑: Cellular senescence[#], inflammation[#] and oxidative stress*

NP polymer type, size, concentration and main findings. In bold, values that were carried across in the assessment of different parameters besides cell viability. *Based on ROS production. [#]Based on interpretation of mRNA levels and protein levels. [f]Based on respiratory levels and ATP production. [g]Based on decrease in NPT levels together with CAT and GST activities. A-PS = Amine-modified Polystyrene. CAT = Catalase. C-PS = Carboxyl-modified Polystyrene. GST = Glutathione S-transferase. LD50 = Median Lethal Dose. MMP = Mitochondrial Membrane Potential. PE = Polyethylene. uPS = unmodified Polystyrene.

10. Summary of connective tissue cell line studies

Authors	Year	Country	Cellular line	Polymer type	NP size (nm)	NP concentration	Exposure duration	Main findings
**Almeida, M. et al.**	2019	Portugal	SAF-1	uPS	100	**0.001, 0.01, 0.1, 1** and **10** mg/L	24h	↑: Oxidative stress[h]<br>=: Cell viability
**Basini, G. et al.**	2022	Italy	ASCs	uPS	100	**5, 25** and **75** μg/mL	24-72h	↓: Cell viability (72h, >5 μg/mL)<br>↑: Oxidative stress and inflammation<br>=: Cell proliferation and ATP levels
**Giannandrea, D. et al.**	2024	Italy	MC3T-E1	uPS	50	1, 10, **50, 100** and 200 μg/mL	4-96h	↓: Cell viability (>100 μg/mL), migratory ability (100 μg/mL) and bone deposition[#] (100 μg/mL)<br>↑: Oxidative stress* (>100 μg/mL), apoptosis[#] (>100 μg/mL) and bone resorption[#] (100 μg/mL)
			MLOY-4					↓: Cell viability (>100 μg/mL)<br>↑: Oxidative stress* (>100 μg/mL), apoptosis[#] (200 μg/mL) and inflammation[#] (100 μg/mL)
**Lin, P. et al.**	2022	China	H9C2	uPS	≈ 94	**30** μg/mL	36h	↑: Oxidative stress* and autophagy<br>Others: Activation of the TGF-β1/Smad signaling pathway
**Poma, A. et al.**	2019	Italy	Hs27	uPS	100	**5, 25,** and **75** μg/mL	4, 24, and 48h	↓: Cell proliferation (48h, 75 μg/mL)<br>↑: DNA damage (>25 μg/mL)<br>=: Oxidative stress*
**Yang, M. et al.**	2022	China	ZF4	A-PS	100	10, **20**, 50, 100, and 200 μg/mL	1, 3, 6 and 9h<br>Cell viability: 24h	↓: Cell viability, cytoplasmic membrane integrity, lysosomal integrity and MMP<br>↑: Oxidative stress* and apoptosis[#]<br>Others: EC50 of 52.8 μg/mL

NP polymer type, size, concentration and main findings. In bold, values that were carried across in the assessment of different parameters besides cell viability. *Based on ROS production. [#]Based on interpretation of mRNA levels and protein levels. [f]Based on respiratory levels and ATP production. [g]Based on decrease in NPT levels together with CAT and GST activities. A-PS = Amine-modified Polystyrene. CAT = Catalase. C-PS = Carboxyl-modified Polystyrene. GST = Glutathione S-transferase. LD50 = Median Lethal Dose. MMP = Mitochondrial Membrane Potential. PE = Polyethylene. uPS = unmodified Polystyrene.
